# Unlocking High-Throughput Plasma-Catalytic Low-Temperature Oxidation of *n*-Hexane over Single-Atom Ag_1_/MnO_2_ Catalysts

**DOI:** 10.1021/jacsau.4c00826

**Published:** 2024-11-23

**Authors:** Zhiping Ye, Chuang Han, Shulin Yang, Yaolin Wang, Ke Wang, Anton Nikiforov, Jiade Wang, Rino Morent, Nathalie De Geyter, Xuming Zhang, Jun Chen, Mi Yan, Junhua Li, Xin Tu, Pengfei Xie

**Affiliations:** † College of Environment, Zhejiang University of Technology, 18 Chaowang Road, Gongshu District, Hangzhou 310014, China; ‡ College of Chemical and Biological Engineering, Zhejiang University, 866 Yuhangtang Road, Hangzhou 310058, China; § Department of Electrical Engineering and Electronics, University of Liverpool, Liverpool L69 3GJ, U.K.; ∥ Research Unit Plasma Technology, Department of Applied Physics, Ghent University, Sint-Pietersnieuwstraat 41, 9000 Ghent, Belgium; ⊥ Key Laboratory of Fluid Transmission Technology of Zhejiang Province, Zhejiang Sci-Tech University, No. 928 Second Street, Xiasha Higher Education Park, Hangzhou 310018, China; # State Key Laboratory of Silicon Materials, School of Materials Science and Engineering, Zhejiang University, 866 Yuhangtang Road, Hangzhou 310058, China; ∇ State Environmental Protection Key Laboratory of Sources and Control of Air Pollution Complex, Tsinghua University, Haidian District, Beijing 100084, China

**Keywords:** plasma catalysis, single atom catalysts, low-temperature reaction, volatile organic compound, synergistic effect

## Abstract

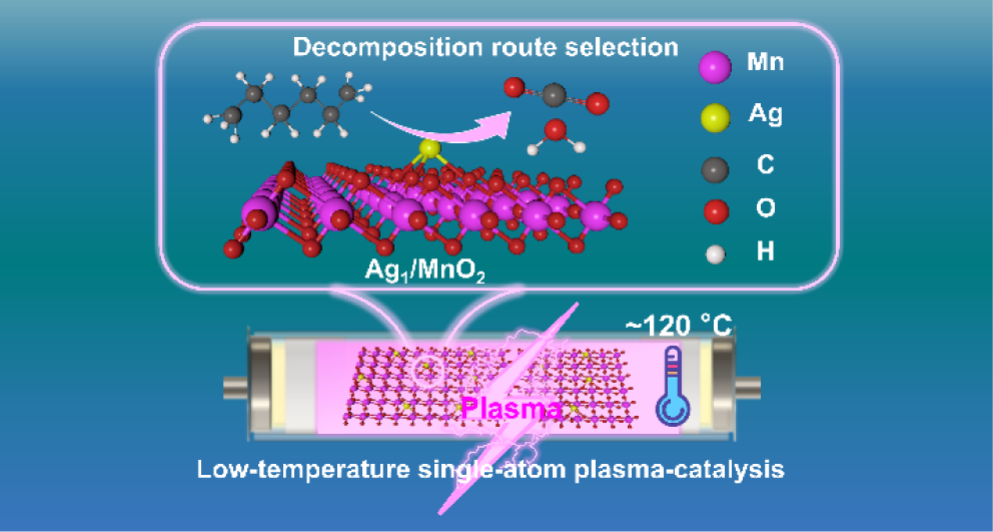

The total oxidation of *n*-hexane, a hazardous volatile organic compound (VOC) emitted by the pharmaceutical industry, presents a significant environmental challenge due to limited catalyst activity at low temperatures and poor stability at high temperatures. Here, we present a novel approach that overcomes these limitations by employing single-atom Ag_1_/MnO_2_ catalysts coupled with nonthermal plasma (NTP). This strategy achieves exceptional performance in *n*-hexane oxidation at low temperatures, demonstrating 96.3% *n*-hexane removal and an energy yield of 74.1 g kW h^–1^ with negligible byproduct formation (O_3_ < 5 ppm, NO_*x*_ < 20 ppm). In situ characterization of the plasma-catalytic system coupled with theoretical calculations revealed a synergistic mechanism for *n*-hexane oxidation. Reactive species generated by the NTP initiate the breakdown of *n*-hexane into smaller fragments. These fragments are then preferentially adsorbed onto the atomic Ag sites due to their favorable energetics, facilitating their subsequent oxidation. The incorporation of single Ag atoms not only enhances the selective adsorption of these NTP-generated intermediates but also accelerates the reaction kinetics. This work demonstrates the potential of single-atom catalysts coupled with NTP for efficient and environmentally friendly removal of VOCs at low temperatures. This approach offers a promising strategy for mitigating industrial air pollution and achieving cleaner air quality.

## Introduction

*n*-Hexane, a prevalent solvent in pharmaceutical production, presents a significant environmental concern due to its extensive use and associated air emissions. Classified as a hazardous air pollutant by the US Environmental Protection Agency, its effective removal presents a critical challenge.^1,2^ Catalytic combustion is a promising process for *n*-hexane removal, particularly when supported by noble metal catalysts. However, the high cost and potential deactivation of these catalysts at elevated temperatures limit their commercial viability.^3^

Single-atom catalysts (SACs) have emerged as promising alternatives, offering tunable active sites and more efficient noble metal utilization.^4−6^ Despite their potential, SACs face limitations such as sluggish kinetics of C–H activation at low temperatures and limited stability at elevated temperatures due to high surface free energy.^7^ Thus, achieving efficient and stable *n*-hexane removal at low temperatures by using SACs remains a significant challenge.

Nonthermal plasma (NTP) provides a promising solution for low-temperature hydrocarbon activation due to its ability to generate high-energetic electrons and reactive species.^8^ These energetic electrons collide with gas molecules, generating a variety of reactive species, such as radicals, ions, and excited molecules, which can efficiently initiate and sustain oxidation reactions under mild conditions. This unique characteristic of NTP makes it particularly attractive for processes like hydrocarbon activation, where traditional thermal methods often require elevated temperatures, leading to higher energy consumption and the risk of catalyst deactivation. However, despite these advantages, the practical application of NTP in *n*-hexane oxidation is currently constrained by low product selectivity and the formation of undesirable byproducts, including ozone (>100 ppm) and NO_*x*_ (>200 ppm), even in the presence of a conventional catalyst.^2,9^

Integrating single-atom catalysts (SACs) with NTP presents a potential solution to address these challenges. Unlike thermal catalysis, NTP overcomes the thermodynamic limitations of high-temperature activation, allowing SACs to function under mild conditions that prevent metal site aggregation.^10,11^ Additionally, the rational design of SACs can selectively capture plasma-generated intermediates for subsequent oxidation to CO_2_, potentially addressing the selectivity issues associated with NTP.^12^ While some studies have explored SACs for various catalytic processes, their integration with NTP for low-temperature chemical reactions remains largely unexplored.^13^ Furthermore, a comprehensive understanding of the synergistic mechanisms between plasma and SACs in this context is entirely lacking.

Herein, we report a novel strategy for high-throughput *n*-hexane oxidation at low temperatures using single-atom Ag_1_/MnO_2_ catalysts coupled with NTP in a double dielectric barrier discharge (DDBD) reactor. Manganese dioxide (MnO_2_) was selected as the support material due to its ability to cycle through multiple oxidation states (Mn^4+^, Mn^3+^, and Mn^2+^), which is crucial for oxidation reactions. This rapid redox cycling enhances electron transfer and facilitates the activation of reactive oxygen species (ROS), including ozone (O_3_) and atomic oxygen (O). Furthermore, the high capacity of MnO_2_ to decompose ozone not only generates reactive species but also prevents an overaccumulation of ozone, contributing to a controlled reaction environment. Silver (Ag), known for its ability to efficiently generate reactive oxygen intermediates from ozone, significantly enhances the oxidative capacity of MnO_2_.^14^ Ag atoms are highly dispersed on the MnO_2_ surface, maximizing the availability of active sites. SACs offer high catalytic activity due to the unique electronic properties of isolated atoms, which facilitate volatile organic compound (VOC) oxidation with greater efficiency. Additionally, Ag increases the electron conductivity of the catalyst, which is essential for promoting efficient electron transfer processes during VOC oxidation.^15^ Therefore, this combination makes Ag_1_/MnO_2_ a promising catalyst for efficient *n*-hexane oxidation under mild conditions.

This approach demonstrates significantly higher gas hourly space velocities (GHSV = 1,886,250 h^–1^) compared to conventional reactors (10,000–30,000 h^–1^), while achieving exceptional *n*-hexane removal and energy yield with minimal byproduct formation. *In situ* characterization and theoretical calculations reveal a synergistic mechanism: plasma-generated reactive species initiate *n*-hexane fragmentation, with the fragments then preferentially adsorbed on the atomic Ag sites for subsequent oxidation. Our work paves the way for utilizing plasma-activated single-atom catalysts for high-throughput VOC abatement at mild temperatures, while offering insights into the low-temperature oxygen activation mechanisms in this process.

## Results and Discussion

### Characterization of Ag_1_/MnO_2_

**Figure 1:**
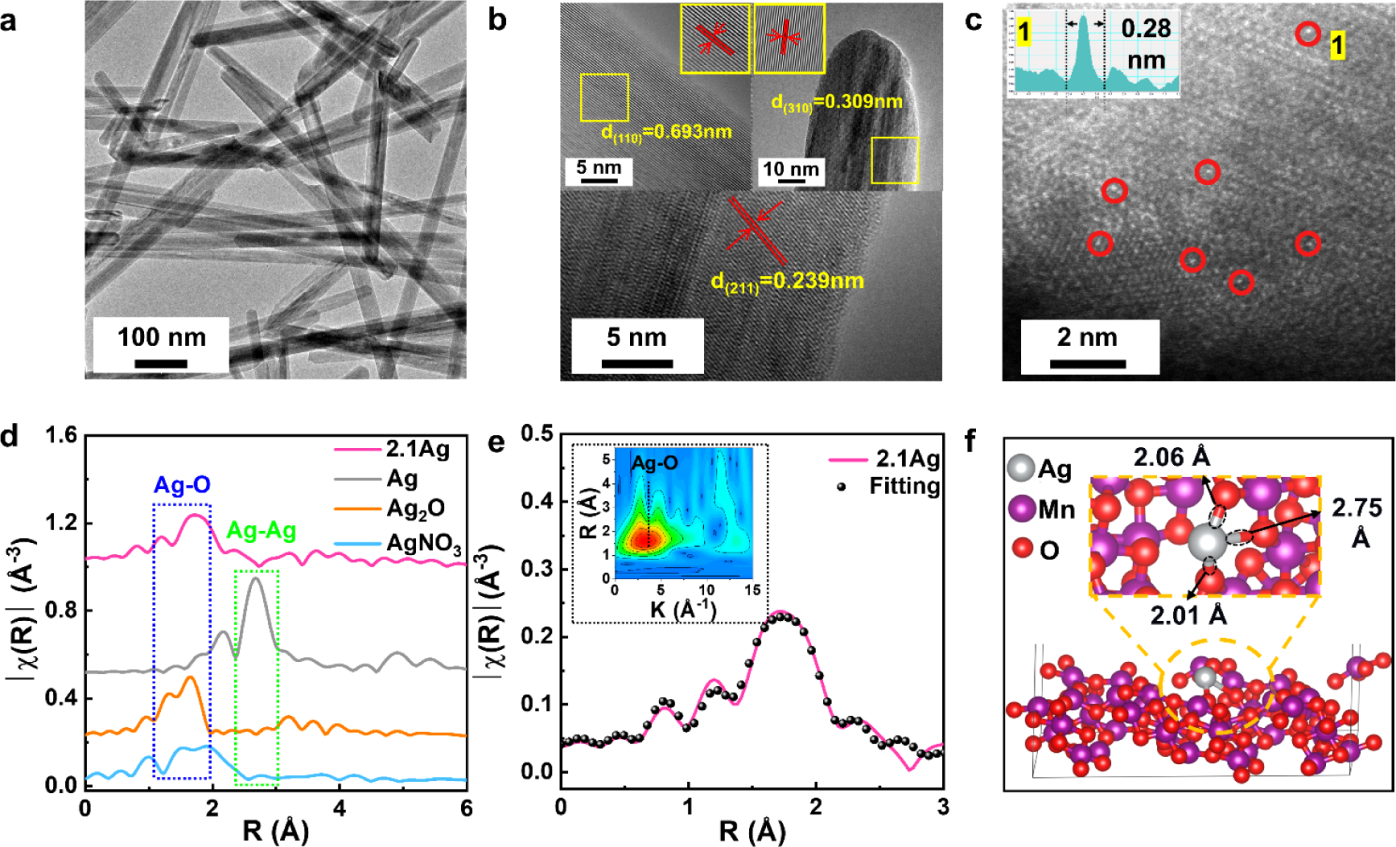
Geometrical characterization of Ag_1_/MnO_2_. (a) TEM and (b) HRTEM images of MnO_2_; (c) HAADF-STEM images of 2.1Ag; (d) EXAFS spectra of 2.1Ag and reference samples; (e) the Ag K-edge R space EXAFS fitting results of 2.1Ag and (f) schematic illustration of the Ag–O moiety structure.

Ag/MnO_2_ catalysts with different Ag loadings (0.7, 2.1, and 7.1 wt %, demoted as *x*Ag, where *x* represents the Ag loading) were synthesized using a hydrothermal method.^16^ The Ag loadings were confirmed by using inductively coupled plasma-atomic emission spectroscopy (ICP-OES). Transmission electron microscopy (TEM) images (Figures 1a and S1a–c) show that both pure MnO_2_ and Ag/MnO_2_ have similar morphologies with an overall nanorod shape (average length of 472 ± 23 nm and average diameter of 34.9 ± 17.3 nm). High-resolution TEM (HRTEM) images of MnO_2_ (Figure 1b) reveals lattice spacings of 0.693, 0.309, and 0.239 nm corresponding to the (110), (310), and (211) planes of MnO_2_, respectively. Interestingly, the Ag_1_/MnO_2_ catalysts do not exhibit any significant differences in their lattice fringes (Figure S1d–i), suggesting that Ag nanoparticles (NPs) might not have formed on the MnO_2_ support in Ag_1_/MnO_2_. Notably, the lattice spacings of these planes increase with Ag doping. Figure S2a shows no diffraction peaks for Ag species like metallic Ag or Ag_2_O, confirming the highly dispersed nature of incorporated Ag, as seen in TEM images (Figure S1a–c). Furthermore, the XRD peaks shift to lower Bragg angles with an increasing Ag loading (Figure S2a). Figure S2b shows that increasing Ag loading increases the normalized intensity of the (220) and (310) planes, while weakening the (211), (301), and (411) planes, consistent with previous reports.^17^ This lattice distortion indicates Ag doping within the MnO_2_ lattice,^18,19^ as supported by TEM observations (Figure S1). Surface area and total pore volume measurements (Table S1 and Figure S3) show no significant differences between Ag_1_/MnO_2_ catalysts, suggesting that Ag loading has a limited impact on these structural properties.

The energy dispersive spectrometer (EDS) mapping images of Ag, Mn, and O on the surface of 2.1Ag (Figure S4) reveal highly dispersed Ag species on MnO_2_. Furthermore, high-angle annular dark-field scanning transmission electron microscopy (HAADF-STEM) images in Figure 1c show Ag atoms (marked in red circles) appearing brighter than Mn due to the difference in elements in Z contrast. These bright dots of Ag atoms (≤0.28 nm in size) are observed only in samples with low Ag loading (0.7 and 2.1Ag, Figure S5a,b). When the Ag loading increases to 7.1 wt %, Ag is primarily present in clusters (marked in red circles, ≤3 nm in size), as shown in Figure S5c. For 2.1Ag, line-profile scanning across three Ag dots confirms their atomic dispersion (insets of Figures 1c and S5b).

Following morphological analysis, a detailed chemical characterization was conducted. First, the coordination of incorporated Ag was investigated by analyzing Fourier transforms of *k*^2^-weighted extended X-ray absorption fine structure (EXAFS) spectra. Figure 1d presents the spectrum for the 2.1Ag sample alongside reference spectra of Ag, Ag_2_O, and AgNO_3_. Notably, the EXAFS spectrum of the 2.1Ag sample exhibits a characteristic peak at 1.7 Å, closely resembling that of the Ag_2_O reference. This suggests the presence of oxidized silver (Ag–O bonds) in 2.1Ag.^20^ Importantly, the peak associated with Ag–Ag scattering at 2.7 Å, observed in the Ag foil spectrum, is absent in 2.1Ag. To further quantify the coordination environment of the single atom Ag, a least-squares EXAFS fitting was performed. As shown in Figure 1e, the fitting curve (black dots) closely matches the experimental data (red line) within the radial distance range of 0–3 Å. A density functional theory (DFT) model, based on Ag coordinated with the (311) plane of MnO_2_, was used for EXAFS fitting. It was found that the coordination number of Ag–O is determined to be 3 in the 2.1 sample, indicating that each atom Ag coordinates bonds with three lattice-bound O atoms. The corresponding Ag–O distances extracted from the fitted theoretical model are 2.01, 2.06, and 2.75 Å (Figure 1f and Table S2). The stability provided by these Ag–O bonds ensures that these sites remain highly active and accessible during plasma-catalytic processes, unlike aggregated Ag clusters, which tend to exhibit lower activity.

**Figure 2:**
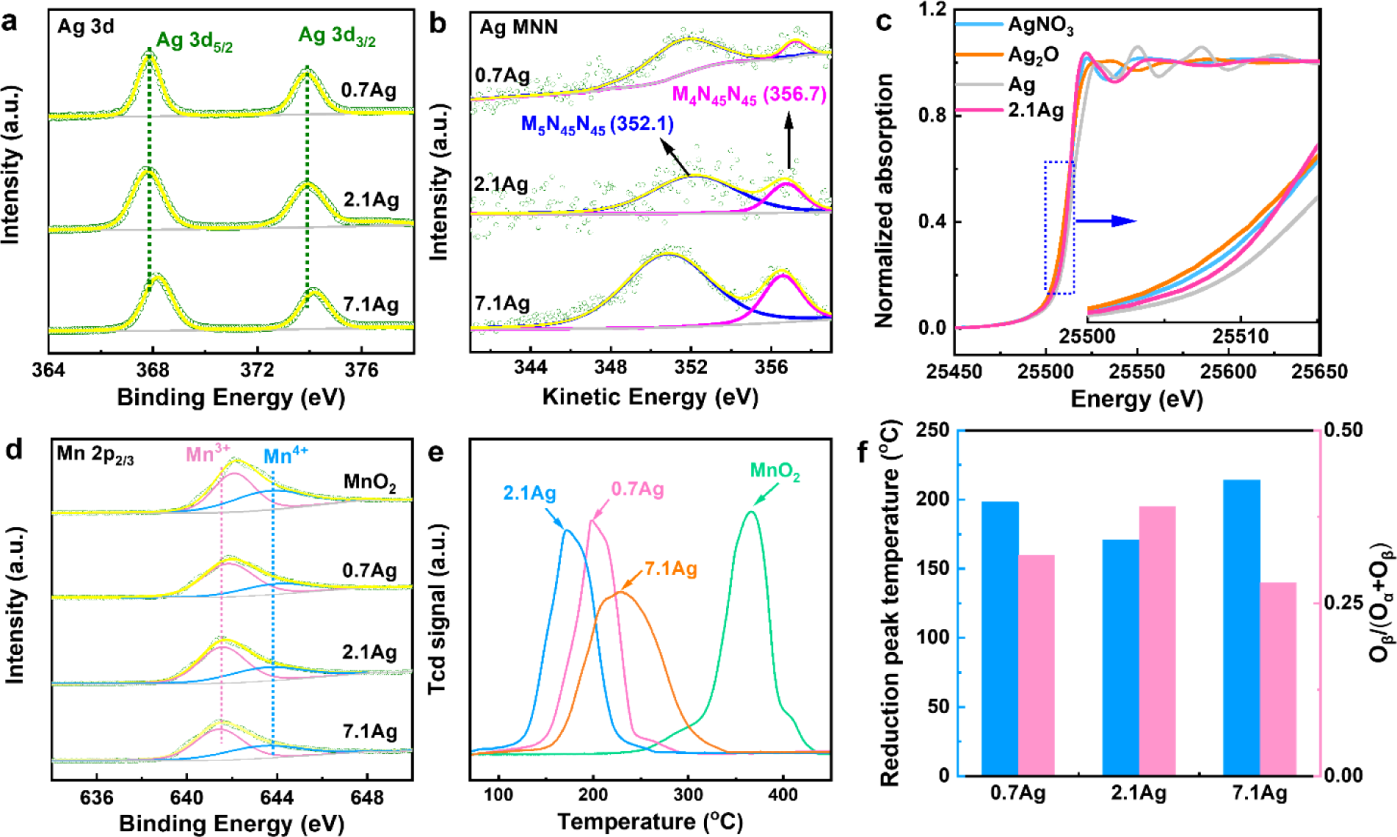
Chemical characterization of Ag_1_/MnO_2_. (a) High-resolution XPS Ag 3d spectra and (b) Ag MNN Auger spectra of Ag-doped samples; (c) Ag K-edge XANES profiles of 2.1Ag and reference samples; (d) high-resolution XPS Mn 2p_2/3_ spectra of MnO_2_ and Ag-doped samples; (e) H_2_-TPR profiles of MnO_2_ and Ag-doped samples; and (f) reduction peak temperature and O_β_/(O_α_ + O_β_) ratio of Ag-doped samples.

High-resolution Ag 3d X-ray photoelectron spectroscopy (XPS) analysis was conducted for the Ag-loaded samples to determine their chemical states (Figure 2a). The Ag 3d doublet appear at binding energies (BE) of 368.0 ± 0.1 eV and 374.0 ± 0.1 eV, with a spin–orbit separation energy of 6.0 eV, corresponding to Ag 3d_5/2_ and Ag 3d_3/2_, respectively.^21^ As Ag loading increases, these peaks slightly shift toward higher BE values (Figure 2a and Table S3), indicating a more cationic character for Ag species in samples with higher Ag loading.^22^ The Auger Ag MNN kinetic energy (KE) region for the Ag-loaded samples (Figure 2b) reveals the presence of M_4_N_45_N_45_ at 356.7 ± 0.1 eV and M_5_N_45_N_45_ at 352.1 ± 0.1 eV. Table S3 summarizes the Auger parameter α_0_ calculated from both photoelectron and Auger peak positions. The α_0_ value of 724.7 eV for 2.1Ag (α_0_ = BE (Ag 3d_5/2_) + KE (M_4_M_45_M_45_)) indicates the presence of oxidized Ag species,^23^ consistent with the EXAFS results (Figure 1e). Further insights into Ag valence were obtained by using X-ray absorption near-edge structure (XANES) spectroscopy (Figure 2c). The XANES spectra of 2.1Ag show a pre-edge transition positioned between the metallic state (Ag^0^) and the oxidation states (Ag_2_O or AgNO_3_), suggesting an oxidation state between 0 and +1 for atomically dispersed Ag on 2.1Ag.^24^ The XANES spectra (Figure 2c) reveal the coexistence of Ag° and Ag^+^, suggesting redox flexibility of the catalysts. This flexibility is crucial for oxygen adsorption and activation, which are key steps in plasma catalytic oxidation reactions.

High-resolution Mn 2p_3/2_ XPS spectra were collected to analyze the chemical state of Mn (Figure 2d). For pure MnO_2_, the Mn 2p_3/2_ spectrum exhibits two peaks at BE values of 642.1 and 643.7 eV, corresponding to Mn^3+^ and Mn^4+^ present in MnO_2_, respectively. However, upon Ag doping, these peaks shift to lower binding energies (Table S4),^25^ which can be attributed to electron transfer from Ag to Mn, driven by the higher electronegativity of Ag (1.93) compared to Mn (1.55), resulting in a lower Mn oxidation state.^26^ This finding can be further confirmed by quantitative analysis of the Mn 2p_3/2_ spectra, which allows for the calculation of the Mn^3+^/(Mn^3+^ + Mn^4+^) ratio (Table S4). Compared to pure MnO_2_, all Ag-doped samples show a higher ratio of Mn^3+^/(Mn^3+^ + Mn^4+^) on the surface. Notably, 2.1Ag exhibits the highest Mn^3+^/(Mn^3+^ + Mn^4+^) ratio. The presence of abundance low-valence Mn and the coordination of lattice oxygen (O_α_) with atomic Ag on the surface of 2.1Ag potentially facilitates the formation of more oxygen vacancies (O_β_), considering the reaction of Mn^4+^ with O^2–^ to generate oxygen vacancies, Ag^+^, and Mn^3+^ to maintain charge balance.^27,28^

The high-resolution spectra of all samples (Figure S6) were also examined. In pure MnO_2_, the spectrum can be deconvoluted into three peaks corresponding to different oxygen species: lattice oxygen (O_α_), oxygen vacancies (O_β_), and surface hydroxyl oxygen (O_h_) at binding energies of 529.2, 531.2, and 533.4 eV, respectively. Interestingly, the O_α_ peak shifts from 529.2 eV in MnO_2_ to a higher BE of 529.4 eV in 2.1Ag. This shift can be attributed to the higher electronegativity of Ag compared to Mn, suggesting the formation of new oxide species, such as Ag–O.^29^ Furthermore, an analysis of the O 1s spectra allowed us to determine the O_β_/(O_α_ + O_β_) ratio for all samples (Table S3). This ratio reflects the concentration of surface oxygen vacancies following the order: 0.39 (2.1Ag) > 0.32 (0.7Ag) > 0.28 (7.1Ag).

In addition, the redox properties of the catalysts were investigated by using hydrogen temperature-programmed reduction (H_2_-TPR), as depicted in Figure 2e. Interestingly, the Ag-loaded samples show a notable shift in the reduction peak to lower temperatures. This shift is most significant for 2.1Ag, followed by 0.7Ag and 7.1Ag, suggesting 2.1Ag has the highest reactivity of lattice oxygen at low temperatures. For clarity, Figure 2f provides the exact TPR peak temperatures and the calculated O_β_/(O_α_ + O_β_) ratio for all samples, further highlighting the superior redox reactivity of 2.1Ag. This enhanced reactivity suggests a potential advantage for selectively adsorbing oxygen species generated during the plasma process.^30^

### High-Throughput Plasma-Catalytic Oxidation of *n*-Hexane

**Figure 3:**
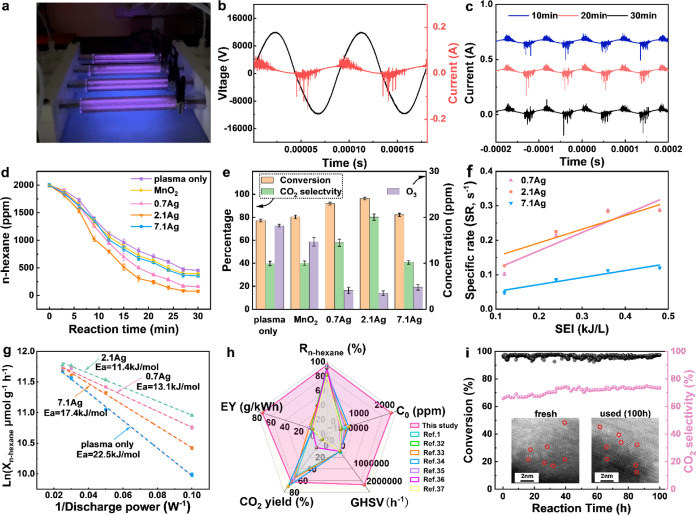
Reaction performance in high-throughput plasma-catalytic oxidation of *n*-hexane. (a) Image of the parallel DDBD reactor in operation; (b) current–voltage waveforms of the discharge over 2 periods; (c) evaluation of the current waveform over operation time (with artificial current waveform shift for better visibility); (d) concentration of *n*-hexane as a function of reaction time; (e) *n*-hexane conversion, CO_2_ selectivity, and ozone concentration for plasma alone and Ag-loaded catalysts; (f) specific rate (SR) of the plasma-catalysis systems as a function of SEI; (g) the molar amount of *n*-hexane converted per unit catalyst (*X*_*n*-hexane_) vs 1/discharge power for the plasma-catalysis systems; (h) comparison of this study and other reported catalytic DBD reactors for *n*-hexane removal; (i) stability test using plasma + 2.1Ag for 100 h.

Figure 3a shows the plasma catalysis reactor operating stably even under high-throughput conditions (gas hourly space velocity (GHSV) of 1,886,250 h^–1^). Figure 3b plots the current–voltage waveforms of the discharge with a specific energy input (SEI) of ∼456 J L^–1^. The burst-peak mode observed in the current waveforms indicates the formation of multiple streamers during the discharge, a characteristic behavior for DBDs.^31^ Notably, the current waveforms remain consistent after 30 min of operation with the 2.1Ag catalyst (Figure 3c), demonstrating the stability of the reaction process. Additionally, the reactor temperature plateaus at ∼120 °C after 15 min of operation with the 2.1Ag catalyst (Figure S7a), further highlighting the stable operating conditions.

Figure 3d shows the degradation of *n*-hexane with increasing plasma reaction time (up to 30 min) under different conditions: plasma alone, plasma with MnO_2_, and plasma with different Ag/MnO_2_ catalysts (0.7, 2.1, and 7.1Ag). For all cases, the *n*-hexane concentration decreases with time, but Ag/MnO_2_ catalysts clearly improve *n*-hexane conversion compared to plasma alone. Figure 3e shows that the Ag_1_/MnO_2_ catalysts achieve higher *n*-hexane conversion compared to plasma alone or plasma with a MnO_2_ support. The 2.1Ag catalyst reaches 96.3% *n*-hexane conversion after 30 min (Figure 3e), significantly exceeding the conversion achieved with plasma alone (77.2%). Notably, higher Ag loading (7.1 wt %) reduces conversion to 82.2%, suggesting optimal performance at a 2.1 wt % loading. In addition, thermal catalytic oxidation of *n*-hexane was conducted using 2.1Ag alone at ∼120 °C. The results showed that the catalyst was unable to be activated for *n*-hexane oxidation at this temperature (Figure S7b).

The CO_2_ selectivity follows the same trend as conversion (Figure 3e), with the 2.1Ag catalyst exhibiting the highest selectivity. This trend further confirms the superior performance of single-atom Ag compared to clustered Ag (7.1 wt %) in promoting *n*-hexane conversion to CO_2_. The significant decrease in organic intermediates with the 2.1Ag catalyst (5.7% compared to 32.5% for plasma alone) confirms its enhanced oxidation ability (Figure S8). Importantly, the plasma-catalytic process effectively minimizes the formation of inorganic byproducts like NO_2_ and ozone compared to plasma alone (Figures 3e and S9). The 2.1Ag catalyst again demonstrates the most significant reduction in byproducts, aligning perfectly with the observed trends in conversion, CO_2_ selectivity, and organic intermediate formation.

Plasma-catalytic oxidation of *n*-hexane was also conducted at lower SEI values. Figure 3f shows that the specific rate (SR) increases with increasing SEI values, demonstrating higher energy input facilitates the catalytic reaction rate at low temperatures. Interestingly, the SR values for the 0.7Ag and 2.1Ag catalysts remain very similar throughout the reaction process, likely due to the presence of atomic Ag sites in both catalysts. In contrast, the SR values for the 7.1Ag catalyst are significantly lower, suggesting that the presence of Ag clusters limits its catalytic activity. These results demonstrate that atomic Ag sites offer enhanced plasma-catalytic conversion of *n*-hexane compared to their clustered counterparts.

Arrhenius plots were used to analyze the apparent activation energy (*E*_a_) for *n*-hexane oxidation in plasma alone and during the plasma-catalytic process (Figure 3g). The plots show the logarithmic reaction rate [ln(*R*_*n*-hexane_)] as a function of the inverse of discharge power (1/discharge power). Compared to plasma alone, the plasma coupled with Ag-loaded catalysts exhibits significantly lower *E*_*a*_ values. Notably, the 2.1Ag sample displays the lowest *E*_a_ value (11.4 kJ mol^–1^). These results again indicate a clear synergistic effect between plasma and Ag_1_/MnO_2_ catalysts. Moreover, a higher loading of single Ag atoms leads to a lower reaction barrier for *n*-hexane oxidation. Figure 3h highlights the excellent performance of the plasma-2.1Ag system compared to previously reported works,^1,32−37^ in terms of *n*-hexane removal (*R*_*n*-hexane_), energy yield (EY), CO_2_ yield (), and GHSV at a similar initial *n*-hexane concentration (*C*_0_), demonstrating the great potential of plasma catalysis for *n*-hexane oxidation on Ag_1_/MnO_2_. At a high-throughput condition of 1,886,250 h^–1^ and an initial *n*-hexane concentration of 2000 ppm, the plasma coupled with 2.1Ag achieved a *n*-hexane conversion of 96.3%, a CO_2_ yield of 77.2%, and the highest energy yield of 74.1 g kW^–1^ h^–1^, which is ∼5.6 times higher compared to using plasma only.

We further evaluated the performance of the 2.1Ag catalyst over an extended period (100 h) for plasma-catalytic *n*-hexane oxidation (Figure 3i). The 2.1Ag catalyst shows a remarkably stable *n*-hexane conversion of ∼96.0% throughout the entire test duration. Additionally, this process generated less than 5 ppm of ozone and 20 ppm of NO_2_ over the full 100 h, indicating the minimal production of harmful byproducts. These results highlight the significant potential of the 2.1Ag catalyst due to its excellent activity, selectivity, and remarkable stability. Following the 100-h stability test, the used 2.1Ag catalyst was characterized using N_2_ adsorption–desorption isotherms (Figure S10a,b), XRD patterns (Figure S10c), and HAADF–STEM images (Figure 3f) for comparison with fresh 2.1Ag samples. No significant changes were observed in the crystal phase of the used catalyst compared to the fresh one, as evidenced by the XRD patterns (Figure S10c). In addition, the specific surface area (Table S5) and pore size distribution of both samples remained almost similar. HAADF–STEM images (Figure 3f) further confirmed the stability of atomic Ag on the MnO_2_ surface, showing no significant alterations after continuous exposure to plasma discharge for 100 h. In summary, these characterizations collectively demonstrate the high stability of the 2.1Ag catalyst for *n*-hexane oxidation, maintaining its excellent performance even after extended operation.

### Reaction Pathways in Plasma-Catalytic Oxidation of *n*-Hexane

**Figure 4:**
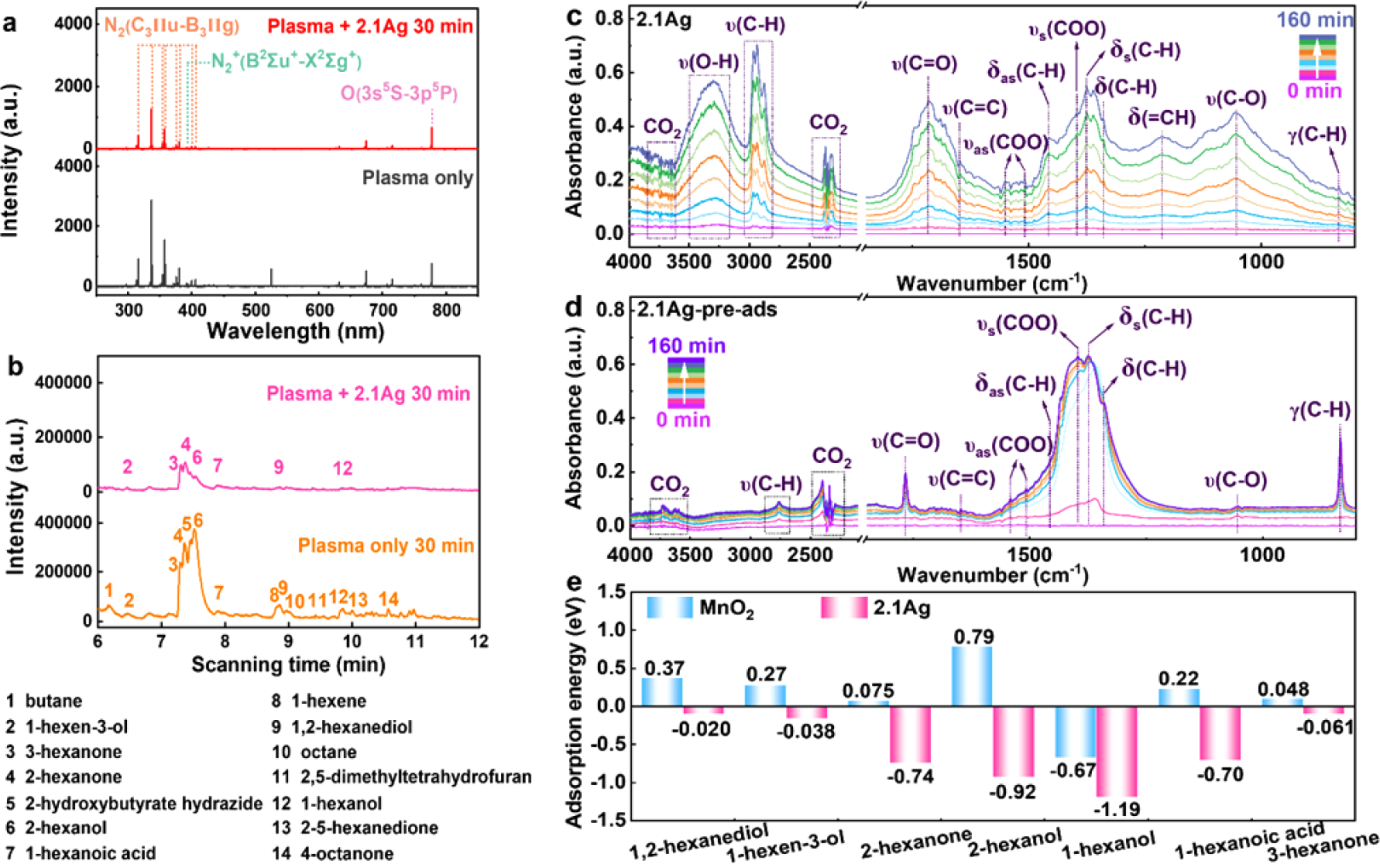
*In situ* characterization of plasma catalytic oxidation of *n*-hexane on Ag_1_/MnO_2_. (a) OES spectra of the discharge generated in *n*-hexane/air using plasma alone and plasma + 2.1Ag; (b) GC–MS spectra of gaseous byproducts of *n*-hexane degradation by plasma alone and plasma + 2.1Ag; (c,d) *In situ* DRIFTS spectra of plasma + 2.1Ag and plasma + 2.1Ag-preads for *n*-hexane degradation (2.1Ag: the 2.1Ag sample was subjected to a plasma discharge in a gas mixture of *n*-hexane and air; 2.1Ag-pread: the 2.1Ag sample with preadsorbed *n*-hexane was subjected to a plasma discharge in air); (e) Adsorption energy diagrams for plasma-catalytic intermediates adsorbed by MnO_2_ and 2.1Ag.

Figure 4a compares the emission spectra obtained using plasma alone and plasma combined with 2.1Ag during the plasma oxidation of *n*-hexane. The spectra reveal the presence of excited states of nitrogen species generated by collisions between high-energy electrons and N_2_ (R1–R4 in Figure S11 and Table S6).^38^ The second positive system (SPS) of N_2_ (C^3^Π_u_–B^3^Π_g_) can be detected in the range of 315–406 nm, while the presence of the first negative system (FNS)  (B^2^–X^2^) with a band head at 391.4 nm suggests the formation of ionic nitrogen molecules. In addition, the O atomic line (3s^5^S–3p^5^P) at 777.4 nm can also be observed in the spectra (R5–R12 in Figure S11 and Table S6).^39^ As shown in Figure S12a,b, the normalized intensities of the second positive system of N_2_ (C^3^Π_u_–B^3^Π_g_) at 337.1 nm are substantially higher for the plasma alone system compared to those with the catalysts (0.7Ag, 2.1Ag, and 7.1Ag). Similarly, the intensity of the O atomic line at 777.4 nm decreases upon addition of the different catalysts (Figure S12c). Since excited N_2_ molecules react with O species to form NO and NO_2_ (R13–16, Figure S11, Table S6), the decreased intensity of plasma species after adding catalysts reduce NO_2_ generation,^40^ as shown in Figure S9. The highly efficient catalytic decomposition facilitated by the single-atom Ag active sites and oxygen vacancies in the 2.1Ag catalyst reduces the availability of atomic oxygen, simultaneously inhibiting the formation of both O_3_ and NO_2_.

**Figure 5:**
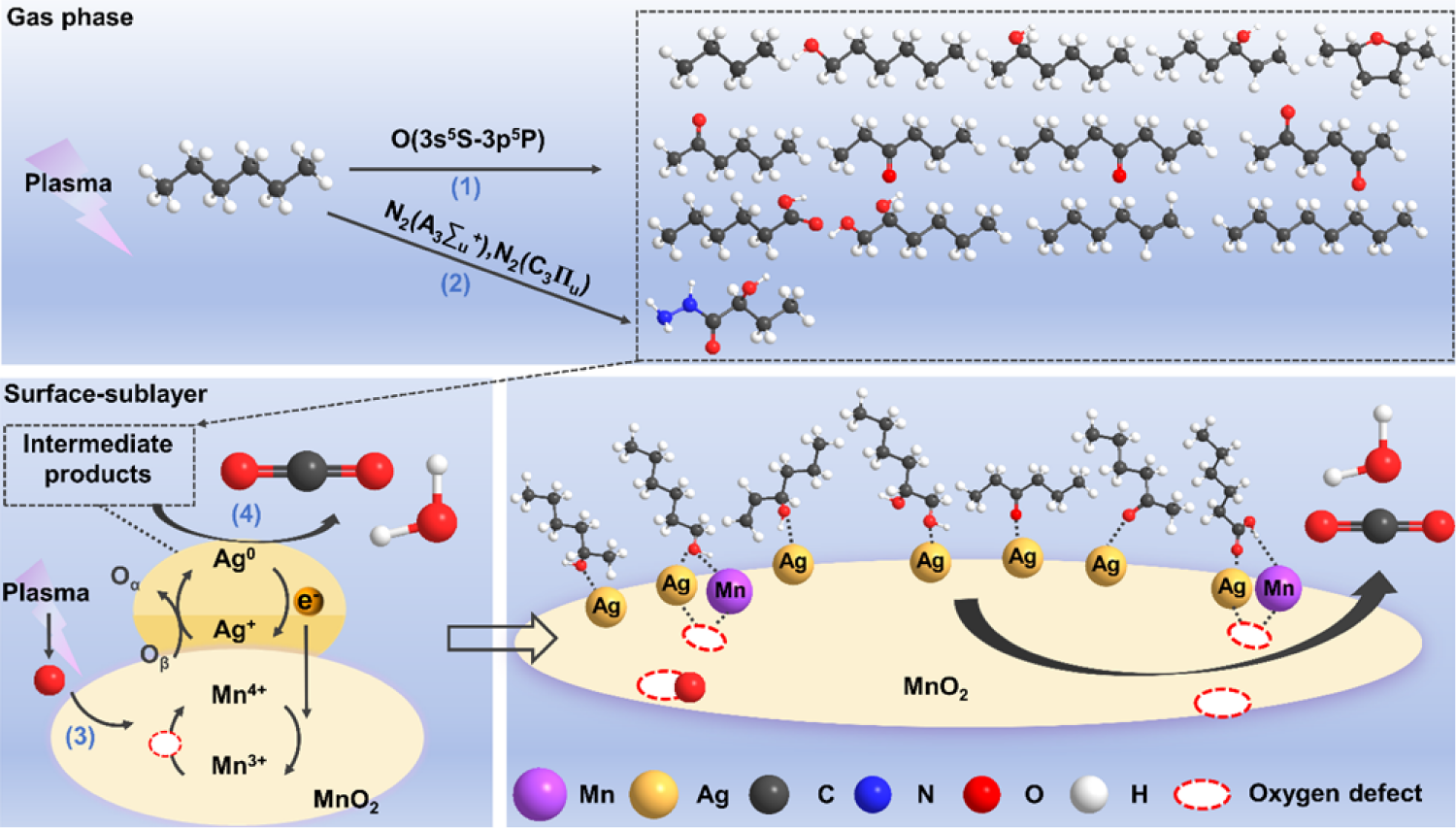
The proposed reaction pathway in the plasma catalytic oxidation of *n*-hexane.

On the other hand, direct collisions between excited species (nitrogen and oxygen) and *n*-hexane are also expected to occur when the plasma is switched on (process 1–2 in Figure 5 and Table S7). These excited species, generated through plasma gas phase reactions (R1–R12 in Figure S11 and Table S6), lead to the formation of oxygenated and nitrogen-containing organic intermediates.^41^ Online GC-MS analysis (Figure 4b) reveals the presence of 14 such organic intermediates (Table S8) during plasma-alone reaction, which can be classified into three categories: hydrocarbons, oxygenated organic compounds, and nitrogenous organic substances. For example, 2-hexanol and 2-hydroxybutyrate hydrazide are produced from the reaction of C_6_H_14_ with these reactive oxygen and nitrogen excited species (process 1–2 in Figure 5 and Table S7).

The introduction of the 2.1Ag catalyst significantly alters the *n*-hexane degradation pathway. As shown in Figure 4b, only half of the initial intermediates remain after 30 min with the catalyst, suggesting the efficient conversion of *n*-hexane. Further GC-MS analysis of the plasma + 2.1Ag system (Figure S13) reveals a decrease in the normalized intensities of specific intermediates (1-hexen-3-ol, 3-hexanone, 2-hexanone, 2-hexanol, 1,2-hexanediol, 1-hexanol, 1-hexanoic acid, and 4-octanone) over time (10 min vs 30 min). This suggests that the 2.1 Ag catalyst not only promotes the oxidation of intermediates to CO_2_ and H_2_O but also accelerates the overall reaction rate, as illustrated in Figure 3.

To elucidate the surface reactions during plasma-catalytic oxidation of *n*-hexane, a customized *in situ* plasma-coupled diffuse reflectance infrared Fourier transform spectroscopic (DRIFTS) system was developed (Figure S14). Figures 4c and S15 present the *in situ* DRIFTS spectra obtained during plasma-catalytic oxidation of *n*-hexane using different catalysts for varying durations (0–160 min). The spectra reveal characteristic peaks of *n*-hexane in the range of 2850–2980 cm^–1^ (ν(C–H)), 1457 cm^–1^ (δ_as_(C–H)), and 1377 cm^–1^ (δ_s_(C–H)),^42,43^ confirming the presence of *n*-hexane on the catalyst surface. The absorption band at 1715 cm^–1^, attributed to the ν(C=O) functional group, indicates the presence of ketones (2-hexanone, 3-hexanone, and 4-octanone) on the catalyst surface during the plasma-catalytic oxidation process. The absorption bands at 1056, 1216, 1377, 1457, 1649, and 3334 cm^–1^ correspond to ν(C–O), δ(=CH), δ_s_(C–H), δ_as_(C–H), ν(C=C), and ν(O–H) respectively,^44,45^ suggesting the presence of 1-hexanol (1056, 1377, 1457, and 3334 cm^–1^), 2-hexanol (1216, 1377, 1457, and 3334 cm^–1^), 1,2-hexanediol (1056, 1216, 1377, 1457, and 3334 cm^–1^), and 1-hexen-3-ol (1216, 1377, 1457, 1649, and 3334 cm^–1^) on the catalysts. Figure S15 reveals the presence of additional bands at 1341, 1397, 1506, and 1546 cm^–1^ for the 2.1Ag catalyst compared to MnO_2_. These bands can be assigned to δ(C–H), δ_as_(COO), and δ_s_(COO),^43,46^ respectively, indicating the presence of hexanoic acid on 2.1Ag. This suggests further oxidation of alcohols and ketones to hexanoic acid over the 2.1Ag catalyst. Additionally, clear peaks in the wavenumber range of 2300–2400 cm^–1^ assigned to gaseous CO_2_^47,48^ are also visible for the 2.1Ag catalyst, which was not the case for MnO_2_. In combination with the gaseous H_2_O band in the range of 3334–3620 cm^–1^, indicative of water formation, this confirms the occurrence of total oxidation of *n*-hexane/intermediates to CO_2_ and H_2_O on the 2.1Ag surface.

To understand the direct adsorption of organic intermediates onto the catalyst surface during the plasma-catalytic oxidation process, *in situ* DRIFTS characterization of the 2.1Ag catalyst preadsorbed with *n*-hexane (2.1Ag-preads) was also conducted under air DBD activation (Figure 4d). The infrared spectrum of 2.1Ag-preads (1216, 2850–2980, and 3620–3850 cm^–1^) is distinctly different from that of the 2.1Ag catalyst without preadsorbed *n*-hexane (Figure 4c). Notably, the absence of surface alcohol intermediates in the 2.1Ag-preads suggests minimal surface reactions to form these intermediates under plasma activation. This contrasting absence of alcohol intermediates on the preadsorbed catalyst further confirms that 2.1Ag actively participates in the adsorption and degradation of intermediates produced by the plasma gas-phase reactions (Figure 4c). Figures 4d and S16 reveal that *n*-hexane preadsorbed on 2.1Ag decomposes under plasma activation, generating intermediate species such as aldehydes (1766 and 2680–2820 cm^–1^), olefins (833, 1216, and 1649 cm^–1^), and carboxylic acids (1397, 1507, and 1541 cm^–1^).^49,50^ This is followed by further oxidation to CO_2_ and H_2_O.^51^ Moreover, a comparison of the *in situ* DRIFTS spectra of preadsorbed MnO_2_ (MnO_2_-preads) and 7.1Ag (7.1Ag-preads) in Figure S17 shows significantly higher CO_2_ production with the 2.1Ag-preads catalyst. This finding is consistent with the observations from other characterizations, suggesting that the single-atom Ag sites on 2.1Ag exhibit stronger redox activity.

Furthermore, the *in situ* DRIFTS experiments with the 2.1Ag catalyst were conducted under thermal activation (Figure S18a). The sample was simply heated to 120 °C (matching the temperature during plasma-catalytic *n*-hexane degradation; Figure S7a) for varying durations (1–12 min). Notably, the DRIFTS spectra (Figure S18a) reveal only *n*-hexane adsorption on the 2.1Ag surface, with no presence of CO_2_ or other intermediates. Additionally, the normalized IR peaks of *n*-hexane (2877, 2936, and 2968 cm^–1^) remain nearly constant (Figure S18b). Combining these observations with the thermal catalytic oxidation results, it becomes clear that a heat-initiated catalytic process is not operative at this low temperature. These findings collectively support the notion that plasma-catalysis on single-atom 2.1Ag catalysts can lower reaction energy barriers, enabling faster oxidation of *n*-hexane and intermediates to CO_2_ under milder conditions.

Figure S16 presents the evolution of various intermediate species over time, as determined by normalized IR intensities for the MnO_2_ and 2.1Ag catalysts. This data clearly demonstrate that the presence of single-atom Ag on the MnO_2_ substrate significantly strengthens the adsorption of all identified intermediates. Density functional theory (DFT) calculations (Figure 4e) support these findings, revealing a preferential adsorption of the seven detected intermediates on the 2.1Ag surface compared to that of MnO_2_. Notably, intermediates like 1-hexanol (−1.19 eV), 2-hexanol (−0.92 eV), 2-hexanone (−0.74 eV), and 1-hexanoic acid (−0.70 eV) exhibit particularly favorable adsorption energies on 2.1Ag. These observations suggest a reaction pathway where the 2.1Ag catalyst effectively adsorbs key intermediates generated by the plasma gas-phase reaction. This selective adsorption of these intermediates facilitates their subsequent surface-catalyzed oxidation.

To elucidate the interaction between adsorbed species and the catalyst surface (e.g., lattice oxygen in Ag–O^2–^–Mn), the used 2.1Ag catalyst was analyzed by XPS and compared to the fresh sample (Figure S19a–c, Table S3). The XPS data reveals an increase in the O_β_/(O_α_ + O_β_) ratio after the plasma-catalytic process, suggesting a decrease in O_α_ (lattice oxygen). This O_α_ consumption suggests its involvement in the oxidation of adsorbed *n*-hexane molecules and intermediates (process 4, Figure 5, Table S7). Meanwhile, the reduction of Mn^4+^ to Mn^3+^ is observed and vacant oxygen sites (O_β_-Mn^3+^) are created (process 3, Figure 5, Table S7). This process is further supported by the observed increase in the Mn^3+^/(Mn^3+^ + Mn^4+^) ratio after plasma activation (Table S4). Additionally, O_2_-temperature-programmed desorption (TPD) profiles of the fresh and used 2.1Ag catalysts (Figure S19d) reveal that the left-shifted peaks at 470, 585, 625, and 725 °C suggest that the 2.1Ag is reduced.^52^ This finding is consistent with the XPS results.

Figure 5 illustrates the proposed reaction pathways for the plasma-catalytic oxidation of *n*-hexane. Plasma discharge generates reactive species that directly react with *n*-hexane in the gas phase, forming intermediate products alongside CO_2_ and H_2_O (process 1–2). The 2.1Ag catalyst then plays a crucial role by selectively adsorbing these plasma-produced intermediates onto its surface. This selective adsorption is crucial for efficient conversion of both the adsorbed intermediates and *n*-hexane molecules that are bound to the catalyst surface through subsequent surface reactions. Furthermore, the plasma discharge generates reactive oxygen species that readily participate in the catalytic process at mild temperatures (process 3–4). These species effectively oxidize the adsorbed intermediates on the 2.1Ag catalyst, significantly enhancing overall reaction efficiency. The presence of Ag_1_/MnO_2_ within the DBD reactor plays a crucial role by promoting the generation of these reactive oxygen species. This ultimately translates to a more efficient conversion of *n*-hexane to CO_2_ through plasma-catalytic oxidation.

## Conclusions

This work investigates the degradation of *n*-hexane using plasma catalysis with Ag_1_/MnO_2_. The combination of plasma with 2.1Ag achieves the highest activity, with a *n*-hexane removal, a CO_2_ selectivity, and an energy yield as high as 96.3%, 80.1%, and 74.1 g kW h^–1^, respectively. In addition, the introduction of the Ag_1_/MnO_2_ catalysts reduces the formation of byproducts such as ozone, nitrogen oxides, and organic intermediates. A detailed investigation reveals that the degradation mechanism in the plasma-catalytic oxidation of *n*-hexane over the Ag_1_/MnO_2_ catalysts mainly involves two steps: (i) direct collisions of high-energy electrons with *n*-hexane molecules and (ii) direct interactions of oxygen and excited nitrogen species with gaseous pollutants. The single Ag atom plays an important role in the adsorption of gaseous intermediates and the highly reactive oxidation of *n*-hexane and intermediates from the plasma reaction. The excited oxygen species produced from the plasma are crucial for subsequent oxidation on the catalyst surface. The fundamental understanding obtained in this work thus paves the way for rational design of efficient single atom catalysts to eliminate volatile organic compounds.

## Supporting Information Available

Experimental details, gas analysis, EDS mapping, calculations of carbon balance, in situ DRITFS experiments, BET, XRD, and O_2_-TPD results, supplement of degradation pathway, and the synergistic effect (PDF)
