# Supplementary material for: Unlocking High-Throughput Plasma-Catalytic Low-Temperature Oxidation of n-Hexane over Single-Atom Ag1/MnO2 Catalysts
Source: JACS Au. 2024 Nov 23;5(1):111–20. doi: 10.1021/jacsau.4c00826 (PMC11775692; doi:10.1021/jacsau.4c00826)
Supplement: Supplementary file 1 — au4c00826_si_001.pdf [file au4c00826_si_001.pdf]

Supporting Information

Unlocking High-Throughput Plasma-Catalytic Low-Temperature Oxidation of *n*-Hexane over Single-Atom Ag_1/MnO_2 Catalysts

Zhiping Ye[a, ‡], Chuang Han[a, ‡], Shulin Yang[b], Yaolin Wang[c], Ke Wang[b], Anton Nikiforov[d], Jiade Wang[a], Rino Morent[d], Nathalie De Geyter[d], Xuming Zhang[e], Jun Chen[a], Mi Yan[f], Junhua Li[g], Xin Tu[c, *], Pengfei Xie[b,*]

[a] College of Environment, Zhejiang University of Technology, 18 Chaowang Road, Gongshu District, Hangzhou 310014, China

[b] College of Chemical and Biological Engineering, Zhejiang University, 866 Yuhangtang Rd, Hangzhou 310058, China

[c] Department of Electrical Engineering and Electronics, University of Liverpool, Liverpool L69 3GJ, UK

[d] Research Unit Plasma Technology, Department of Applied Physics, Ghent University, Sint-Pietersnieuwstraat 41, 9000 Ghent, Belgium

[e] Key Laboratory of Fluid Transmission Technology of Zhejiang Province, Zhejiang Sci-Tech University, No. 928, 2nd Street, Xiasha Higher Education Park, Hangzhou 310018, China

[f] State Key Laboratory of Silicon Materials, School of Materials Science and Engineering, Zhejiang University, 866 Yuhangtang Rd, Hangzhou 310058, China

[g] State Environmental Protection Key Laboratory of Sources and Control of Air Pollution Complex, Tsinghua University, Haidian District, Beijing 100084, China

* E-mails: pfxie@zju.edu.cn; xin.tu@liverpool.ac.uk

‡ These authors contributed equally to this work.

Table of Contents:

1. Materials and methods

1.1 Experimental setup

1.2 Catalyst synthesis

1.3 Catalyst characterization

1.4 Optical emission spectroscopy diagnostics

1.5 Density functional theory calculations

1.6 Quantitative analysis of reaction gases or exhaust

1.7 Definition of performance parameters

2. Catalyst characterization

3. Carbon balance and by-products

4. Characterization of used 2.1Ag after a 100-h plasma-catalysis process

5. Supplement of degradation pathway and the synergistic effect

6. References

1. Materials and methods

1.1 Experimental setup

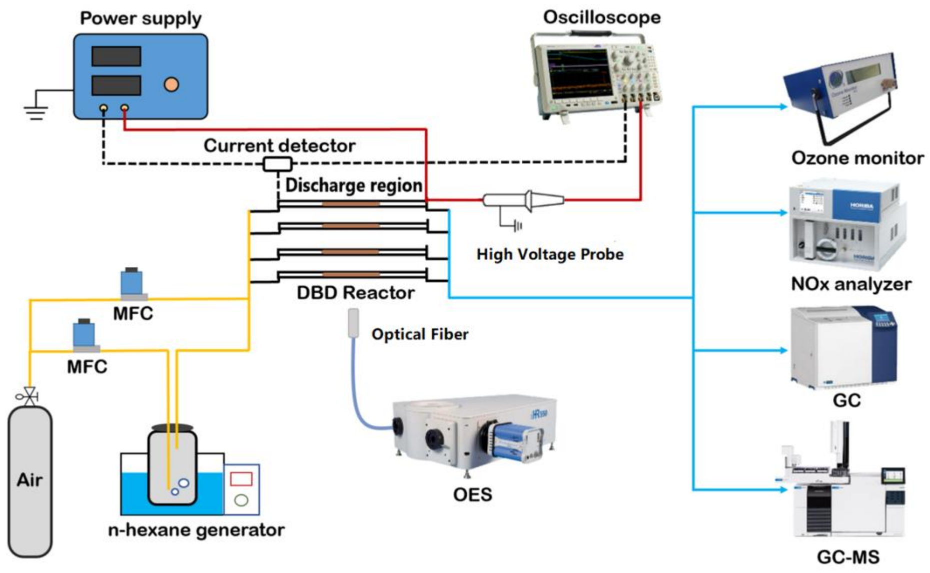

Scheme 1. Experimental set-up for high-throughput plasma-catalytic oxidation of n-hexane.

Scheme 1 shows the experimental set-up consisted of three main parts: the gas supply system, the parallel double dielectric barrier discharge (DDBD) system and the gas analysis system. High-purity air (99.99%, Jingong) was used as carrier gas in the gas supply system. Gaseous n-hexane was generated by bubbling air through a liquid n-hexane (99.99%, Sinopharm) reservoir placed in a water bath maintained at a constant temperature of 298 K. The n-hexane loaded air stream was mixed with pure air before entering the plasma reactor and the total gas flow rate and the initial concentration of n-hexane were set to 5 L min^{-1} and 2000 ppm respectively making use of 2 mass flow controllers (MFCs, CS200-A, Beijing Sevenstar Flow Co., Ltd). In this work, the used four-channel parallel DDBD reactor consists of 4 parallel identical DBDs, which each consists of two coaxial cylindrical quartz tubes, one stainless steel electrode rods acting as high-voltage electrode and one copper mesh operating as grounded

electrode. Each outer quartz tube is 400 mm long (outer diameter (OD): 25 mm, inner diameter (ID): 17 mm), while each inner quartz tube is 485 mm long (OD: 12 mm, ID: 8 mm). The discharge length of each tube is 200 mm (mesh length) and the discharge gap is 2.5 mm. In the discharge zone, approximately 0.2 g of catalysts were packed in each reactor and kept in place by quartz wool, and the gas hourly space velocity (GHSV) is 1,886,250 h^{-1}. The high-voltage rod electrode was connected to an AC plasma generator (PG-2000K, Nanjing Suman Electronic Co.). An oscilloscope was used to measure voltage and current signals (DPO-3012, Tektronix). In addition, the DBD reactor temperature are monitored by an infrared temperature detector (Fluke VT04/VT04A).

1.2 Catalyst synthesis

In a first step, MnO_2 was prepared using the hydrothermal method. 75 mL of an aqueous solution containing 2.484 g $MnSO_4 \cdot H_2O$ (AR, Maclean) and 1.659 g $KMnO_4$ (AR, Sinopharm) was added to a 100 mL Teflon-lined stainless-steel autoclave and heated at 160 °C for 24 h. In a next step, the resulting slurry was washed and dried for 24 h, followed by calcination at 500 ℃ in air for 6 h. The prepared MnO_2 were subsequently also loaded with silver (3.6 wt%, 13.6 wt% and 16.8 wt%) to prepare atomically dispersed Ag/ MnO_2 catalysts. In a first step, an ammonia solution was added to an $AgNO_3$ solution, and then mixed with 30 mL of 30 Vol% H_2O_2 (AR, Sinopharm) and 2 g MnO_2, followed by washing, drying and calcination at 500 °C for 6 h to obtain the desired Ag-loaded samples. To determine the exact elemental composition of the prepared catalysts, inductively coupled plasma-optical emission spectroscopy (ICP-OES) was used (720ES, Agilent). Using this technique, the actual Ag loading on the MnO_2 samples was

found to be 0.7 wt%, 2.1 wt% and 7.1 wt% respectively. Hence, the obtained Ag-loaded catalysts will be denoted as 0.7Ag, 2.1Ag, and 7.1Ag in this study.

1.3 Catalyst characterization

Nitrogen (N_2) adsorption-desorption isotherms were collected at 77 K to determine the specific surface area (SSA), specific pore volume, and average pore diameter using a surface area analyzer (BSD, PS2). The samples were first degassed under vacuum at 200 °C for 6 h. The specific surface area of the catalysts was calculated from the isotherms using the Brunauer-Emmett-Teller (BET) theory. X-ray diffraction (XRD) characterization was carried out on a Bruker D8-advance diffractometer using Cu-Kα radiation (40 kV, 200 mA) from 10 to 80° with a scan speed of 6 ° min^{-1}. Microscopic morphological analysis of the prepared catalysts was performed using scanning electron microscopy (SEM) (JEOL, JSM-7001F), high-resolution transmission electron microscopy (HRTEM) (Hitachi, HT7700) and AC-HAADF-STEM (JEOL, JEM-ARM200F). The scan was conducted at a rate of 2 ° min^{-1}. To gather information on the surface chemical composition of the catalysts, X-ray photoelectron spectroscopy (XPS) analysis was done with a Thermo Scientific K-Alpha XPS apparatus using monochromatic AlK_α X-ray radiation at 250 W. The C 1s photoelectron peak at 284.8 eV was used to reference all binding energies. To evaluate the hydrogen-temperature programmed reduction (H_2-TPR) and oxygen-temperature programmed desorption (O_2-TPD) of the samples, a fully automated programmed temperature rise chemisorption analyzer was used (Bel Cata II, Microtrac).

To measure the metal dispersion (D_{metal}), we employed pulse hydrogen (H_2) chemisorption using a BELCat II instrument (Microtrac). Approximately 1g of the Ag/MnO_2 catalyst (40-60

mesh) was pretreated at 200 °C to remove adsorbed moisture and then reduced under hydrogen flow at 700 °C. After cooling, excess hydrogen was purged using helium. The system was then stabilized, and hydrogen pulses were introduced to quantify the chemisorbed hydrogen, from which the number of exposed Ag atoms (and D_{metal}) was determined.

Ag K-edge X-ray absorption fine structure (XAFS) spectra were recorded in transmission mode at room temperature using the BL14W1 XAFS beam line at the Shanghai Synchrotron Radiation Facility. The X-ray absorption near edge structure (XANES) and extended X-ray absorption fine structure (EXAFS) data reduction and analysis were performed using the Athena program which is part of the inverse fast Fourier transform (IFF) FIT software package. The filtered k2 weighted $\chi(k)$ was Fourier-transformed into the R space (k range: 2.9–14.3 Å^{-1} for Ag-K EXAFS).

We used a JASCO FT/IR-4700 spectrometer coupled with a custom-made in situ cell to monitor the oxidation of n-hexane on the catalyst surface under plasma exposure at various conditions. Three different experiments (A, B, and C) were designed and the procedures are outlined below:

A. Degradation of n-hexane on catalysts in n-hexane/air plasma

i. Catalyst preparation: The MnO_2 and 2.1Ag catalysts were pressed and sieved to obtain particles with a mesh size of 40-60. Approximately 50 mg of the sieved samples were placed in the in situ reaction cell.

ii. Pre-treatment: The catalyst was pre-treated with Ar (300 mL min^{-1}, 99.999% purity) in the in situ reaction cell at 180 °C for 30 min to clean its surface.

iii. Background collection: The in situ reaction cell was cooled to 25 °C, and the IR background was collected after flushing the cell with air (300 mL min^{-1}, 99.999% purity).

iv. Plasma exposure: A mixture of n-hexane bubbled through air (1.5 mL min^{-1}) was introduced, and the plasma was activated (~0.1 W). IR spectra were collected every 20 min over a period of 160 min.

v. Post-reaction: After switching off the plasma, the system was flushed with Ar (300 mL min^{-1}) for 40 min, and the corresponding IR spectra were collected to monitor the decrease of adsorbed peaks.

B. Oxidation of pre-adsorbed n-hexane on catalysts in air plasma

i. Catalyst preparation: The MnO_2 and Ag/MnO_2 catalysts were pressed and sieved (40-60 mesh). Approximately 50 mg of the catalyst was placed in the in situ cell and exposed to ~500 ppm of n-hexane for 30 min.

ii. Stabilization: Ar (300 mL min^{-1}, 99.999% purity) was used to flush the cell for 3 h until the IR-detected n-hexane peaks stabilized, ensuring full adsorption of n-hexane onto the catalyst surface.

iii. Plasma exposure: Air (400 mL min^{-1}, 99.999% purity) was introduced, and the plasma was switched on (~0.1 W). IR spectra were recorded every 20 min for 160 min.

iv. Post-reaction: The plasma was turned off, and Ar (300 mL min^{-1}) was used to purge the system for 40 min, with IR spectra collected during this period to observe desorption.

C. Thermal catalytic oxidation of n-hexane using 2.1Ag catalyst at 120 °C

i. Catalyst preparation: 50 mg of the sieved 2.1Ag catalyst was placed in the in situ reaction

cell and pre-treated with Ar (300 mL min^{-1}, 99.999% purity) at 180 °C for 30 min.

ii. Background collection: The temperature was decreased to 25 °C, and the IR background was recorded after flushing with air (300 mL min^{-1}, 99.999% purity).

iii. Thermal oxidation: An n-hexane/air mixture (1.5 mL min^{-1}) was introduced, and the catalyst was heated to 120 °C. IR spectra were collected at 1, 12, and 30 min.

iv. Post-reaction: After the reaction, the heater was turned off, and Ar (300 mL min^{-1}) was used to flush the system, with IR spectra recorded for 40 min to track the desorption of reaction products.

1.4 Optical emission spectroscopy diagnostics

Optical emission spectroscopy (OES) measurements were performed using a Horiba iHR550 spectrometer, featuring a spectral resolution of ± 0.20 nm. The light from the plasma was collected through an optical fiber positioned a few millimeters from the plasma discharge region to minimize spectral interference and ensure accurate data collection. The optical fiber was aligned perpendicular to the axis of the quartz reactor to avoid signal distortion.

The spectra were recorded across a wavelength range of 250-800 nm, which was selected to capture the emission lines and bands of key reactive species, such as atomic oxygen (O), hydroxyl radicals (OH), nitrogen species (N_2), and other species relevant to plasma-catalytic oxidation. The OES measurements were conducted under steady-state conditions, with the plasma discharge operating at a power level of 30 W and room temperature. These conditions were chosen to mimic the reaction environment used during catalytic performance testing.

The emission spectra of species such as atomic oxygen (O), hydroxyl radicals (OH), and nitrogen species (N_2) were analyzed due to their critical roles in the plasma-catalytic oxidation of n-hexane. Atomic oxygen and OH radicals are known to drive oxidation pathways, while N_2 species contribute to energy transfer mechanisms in the plasma, influencing overall reactivity. To ensure accuracy, the spectrometer was calibrated with a mercury-argon lamp before each measurement. Emission intensities were normalized and compared to reference spectra from the literature. Control measurements without plasma discharge were also performed to account for background emission.

1.5 Density functional theory calculations

Density functional theory (DFT) calculations were conducted to reveal the adsorption properties of the catalysts under study. The calculations were done using the Vienna ab initio simulation package (VASP) employing the projector augmented wave (PAW) approach. The exchange-correlation interaction was described using the van der Waals (vdW) functional, while analysis of the electronic structure was done using the Perdew-Burke-Ernzerhof (PBE) exchange-correlation functional. Standard models were performed to ensure accuracy[1], confirming the optimized lattice constants of bulk MnO_2 through consistency with their corresponding experimental values. To accurately represent the surface characteristics, MnO_2 (310) surface models were constructed using a triple-layer slab model. For the Brillouin zone, a (3 × 3 × 1) k-point mesh was employed. To maintain the overall stability of the system, the bottom layer of the slab model was designated as stationary. The remaining atoms were granted

the freedom to relax until the residual forces diminished below a threshold of 0.02 eV Å^{-1}. The adsorption energy (E_{ads}) for various gas molecules was calculated using the following equation: $E_{ads} = E_{tot} - (E_{gas} + E_{cat})$ where E_{tot}, E_{gas} and E_{cat} are the energies of the adsorption structures, the gas molecules and the catalyst structures respectively.

1.6 Quantitative analysis of reaction gases or exhaust

A gas chromatograph (GC9790, Fuli) with a flame ionization detector (FID) was used to determine the concentration of n-hexane in the in- and outlet of the plasma reactor after 30 min discharge. The same GC device, but equipped with a thermal conductivity detector (TCD), was used to measure the concentrations of produced CO_2 and CO. To determine the O_3 concentration, an ozone monitor (Model 106-MH, 2B Technologies) was connected to the outlet of the plasma reactor. The organic intermediates present in the exhaust gas were detected using a gas chromatograph-mass spectrometer (GC-MS, 7890A-5975C, Agilent), while the nitrogen oxides (NO_2 and NO) present in the exhaust gas were detected using an NO_x analyzer (VA-5000, Horiba).

The experimental details of the GC-MS analysis of the intermediates are outlined below: The GC-MS analysis (7890A-5975C, Agilent) commenced at an initial chamber temperature of 60 °C, maintained for 5 min to ensure thermal stability. The temperature was then ramped at 10 °C min^{-1} to 180 °C for effective separation of early-eluting compounds. Between 180 °C and 210 °C, the ramp rate was reduced to 1 °C min^{-1} to monitor intermediate formation under controlled conditions. The temperature was subsequently increased to 250 °C at 5 °C min^{-1} and

held constant for 5 min to allow complete elution of volatile species.

Exhaust gas samples were immediately collected in a 200 mL gas sampling bag after the reaction to minimize sample loss. They were analyzed promptly for accuracy, with a 1 μL gas injection into the GC-MS system using an autosampler. A 60 m × 0.25 mm × 0.25 μm DB-VRX capillary column provided high-resolution separation of exhaust gas components. Compound identification was achieved by matching retention times and mass spectra with reference data from the NIST/EPA/NIH Mass Spectral Library.

To validate the identification of intermediates, in situ infrared (IR) spectroscopy (NICOLET iS50 FT-IR, Thermo Fisher Scientific) was performed concurrently with the GC-MS analysis. The IR spectra recorded using the 2.1Ag catalyst were compared with literature reference spectra, enhancing the reliability of compound identification by providing complementary confirmation of functional groups and bonding environments in the intermediates.

1.7 Definition of performance parameters

To investigate the activation energy under plasma conditions, we varied the discharge power at levels of 10 W, 20 W, 30 W, and 40 W. The activation energy (E_a) was determined by plotting the natural logarithm of the molar amount of n-hexane converted per unit catalyst against the reciprocal of discharge power (1/P). This approach allows for a linear fit, where the slope corresponds to the activation energy (E_a) in kJ mol^{-1}.

The n-hexane removal ($R_{n\text{-hexane}}$), CO_2 yield (Y_{CO_2}), CO yield (Y_{CO}), CO_2 selectivity

(S_{CO_2}), CO selectivity (S_{CO}), discharge power (P), the specific energy input (SEI), energy yield (EY), specific rate (SR) and the molar amount of n-hexane converted per unit catalyst ($X_{n\text{-hexane}}$) were calculated as follows:

$$\eta_{n\text{-hexane}}\ (\%) = \frac{[\text{n-hexane}]_{in} - [\text{n-hexane}]_{out}}{[\text{n-hexane}]_{in}} \times 100\% \tag{1}$$

$$Y_{CO_2}\ (\%) = \frac{[CO_2]_{out}}{6 \times [\text{n-hexane}]_{in}} \times 100\% \tag{2}$$

$$Y_{CO}\ (\%) = \frac{[CO]_{out}}{6 \times [\text{n-hexane}]_{in}} \times 100\% \tag{3}$$

$$S_{CO_2}\ (\%) = \frac{[CO_2]_{out}}{6 \times ([\text{n-hexane}]_{in} - [\text{n-hexane}]_{out})} \times 100\% \tag{4}$$

$$S_{CO}\ (\%) = \frac{[CO]_{out}}{6 \times ([\text{n-hexane}]_{in} - [\text{n-hexane}]_{out})} \times 100\% \tag{5}$$

$$P\ (W) = \left[\int_0^T U(t)\, I(t)\, dt\right] \times f \tag{6}$$

$$SEI\ (J\ L^{-1}) = \frac{P\ (W) \times 60\ (s\ min^{-1})}{\text{gas flow rate}\ (L\ min^{-1})} \tag{7}$$

$$EY\ (g\ kW^{-1}\ h^{-1}) = \frac{M_{n\text{-hexane}} \times ([\text{n-hexane}]_{in} - [\text{n-hexane}]_{out}) \times Q \times 60}{P\ (W) \times 22.4} \times 10^{-3} \tag{8}$$

$$SR\ (s^{-1}) = \frac{\text{Number of moles of degraded n-hexane (mol } s^{-1})}{D_{metal}\ (\%) \times \text{Number of moles of metal loading (mol)}} \tag{9}$$

$$X_{n\text{-hexane}}\ (\mu mol\ g^{-1}\ h^{-1}) = \frac{\text{Number of moles of degraded n-hexane } (\mu mol\ h^{-1})}{\text{The amount of catalyst used (g)}} \tag{10}$$

$$E_a\ (kJ\ mol^{-1}) = SEI \times Ln(X_{n\text{-hexane}}) = \frac{P \times 60 (s\ min^{-1})}{Q\ (L\ min^{-1})} \times Ln(X_{n\text{-hexane}}) \tag{11}$$

where $[\text{n-hexane}]_{in}$ (ppm) and $[\text{n-hexane}]_{out}$ (ppm) represent the inlet and outlet concentrations of n-hexane; $[CO_2]_{in}$ (ppm) and $[CO_2]_{out}$ (ppm) represent the inlet and outlet CO_2 concentrations; $[CO]_{in}$ (ppm) and $[CO]_{out}$ (ppm) represent the inlet and outlet CO concentrations; U(t) (kV) and I(t) (mA) are the applied voltage and discharge current, respectively; *f* is the frequency (kHz); *T* (ms) is the discharge period; 22.4 (L mol^{-1}) is the molar volume of gas under standard conditions; $M_{n\text{-hexane}}$ (g mol^{-1}) represents the relative molecular mass of n-hexane; Q (L min^{-1}) is the flow rate of the inlet gas and D_{metal} (%) is the Ag dispersion on the catalysts.

2. Catalyst characterization results

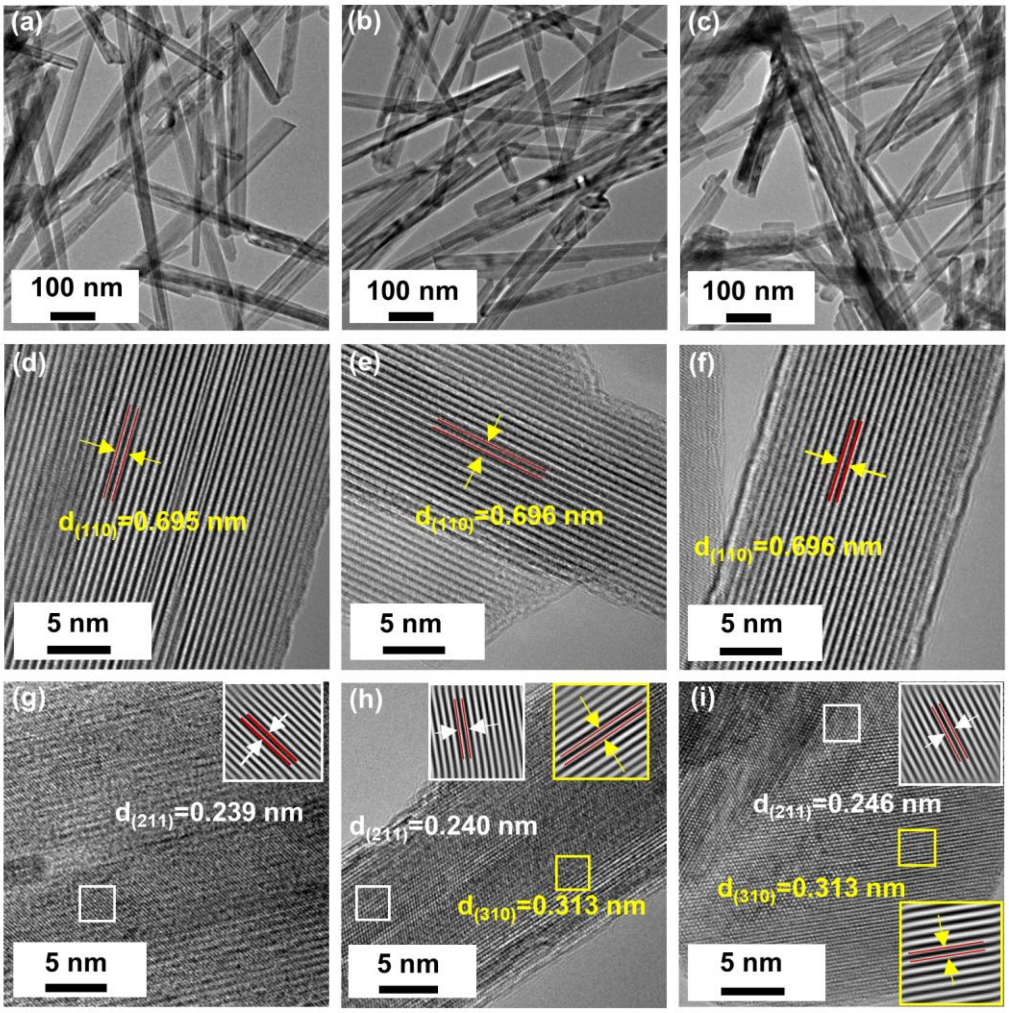

Figure S1. (a-c) TEM images of 0.7Ag, 2.1Ag, and 7.1Ag; (d-f) HRTEM images of the (110) crystal planes of 0.7Ag, 2.1Ag, and 7.1Ag; (g-i) HRTEM images of the (211) and (310) crystal planes of 0.7Ag, 2.1Ag, and 7.1Ag.

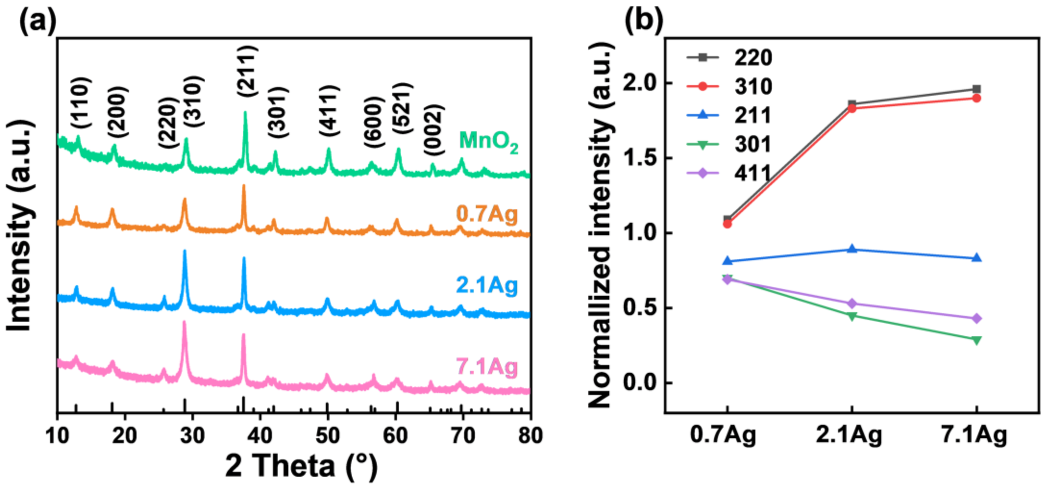

Figure S2. (a) XRD spectra of MnO2, 0.7Ag, 2.1Ag, and 7.1Ag; (b) Normalized intensities of the (220), (310), (211), (301), and (411) planes of 0.7Ag, 2.1Ag, and 7.1Ag.

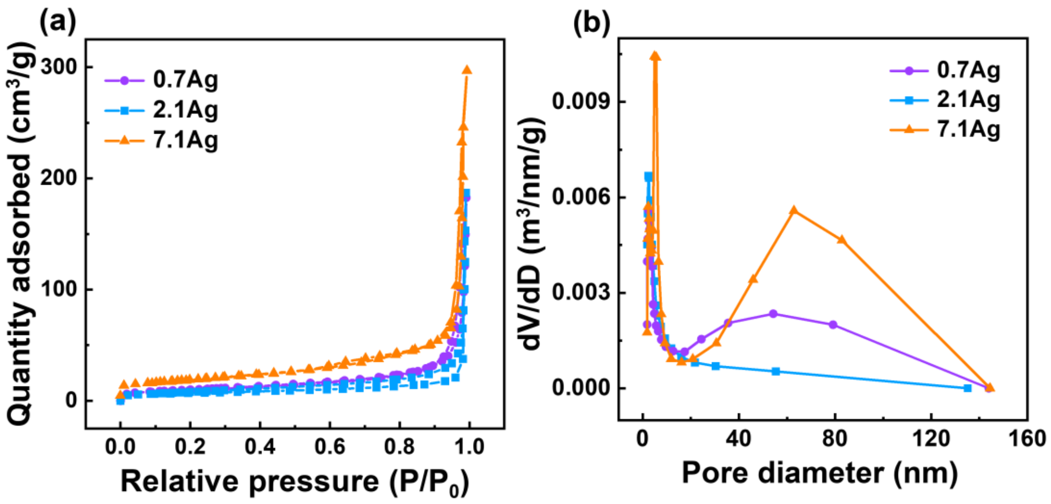

Figure S3. (a) N_2 adsorption-desorption isotherms of 0.7Ag, 2.1Ag and 7.1Ag; (b) BJH pore size of 0.7Ag, 2.1Ag and 7.1Ag.

Table S1. Textural properties of the catalysts.

Sample	Ag loading (wt.%)	S_{BET}[a] (m^2 g^{-1})	D_{pore}[b] (nm)	V_{pore}[c] (cm^3 g^{-1})	Crystallite[d] size (nm)	D_{metal}[e] (%)
MnO_2	-	50.6	22.9	0.6	4.7	-
0.7Ag	0.7	35.8	26.4	0.3	4.2	36.8
2.1Ag	2.1	36.5	24.4	0.3	3.8	13.0
7.1Ag	7.1	36.5	24.5	0.4	4.0	8.3

[a] The BET surface areas are calculated from the N_2 adsorption isotherm obtained at 77 K; [b] D_{pore} = the average pore diameter; [c] V_{pore}= total pore volume at p/p_0 ~0.99, [d] The crystallite size is calculated from the characteristic peak of the (211) crystal plane in the XRD patterns; [e] Ag dispersion (D_{metal}) was measured by hydrogen pulse adsorption.

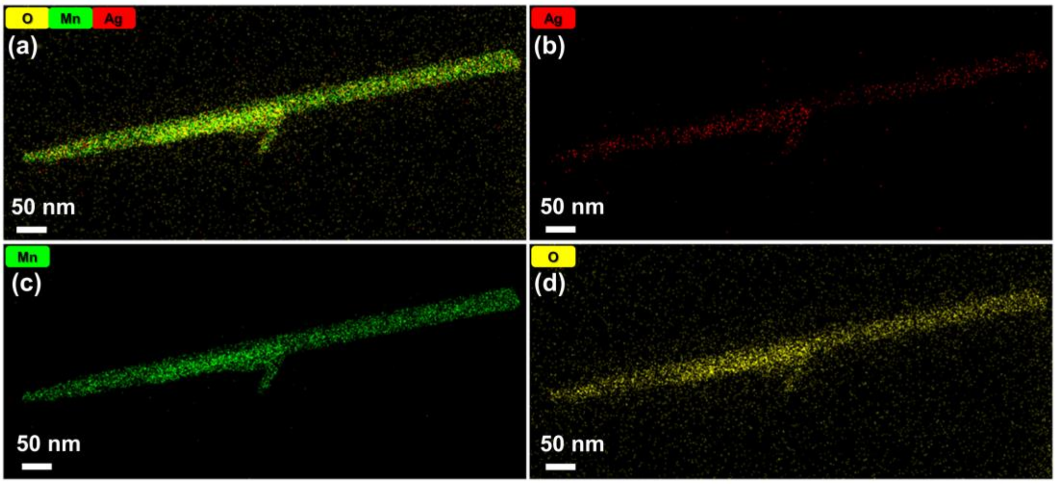

Figure S4. EDS mapping images of the elements Ag, Mn and O on 2.1Ag.

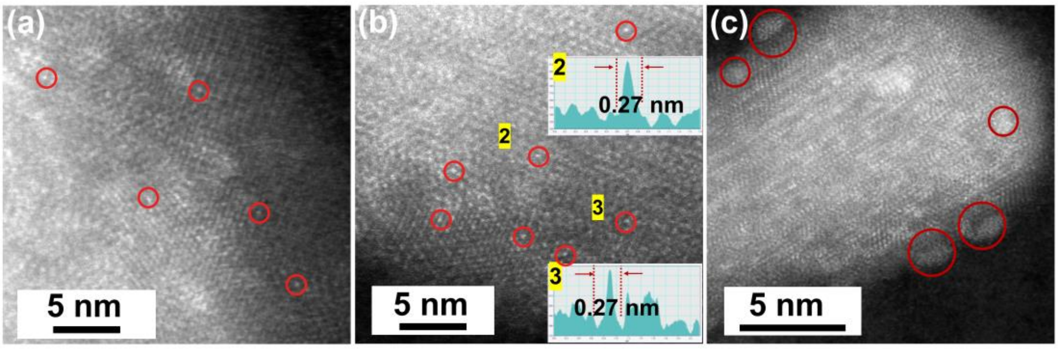

Figure S5. HAADF-STEM images of (a) 0.7Ag, (b) 2.1Ag and (c) 7.1Ag.

Table S2. Fitting results of the EXAFS spectrum of 2.1Ag.

Scattering path	CN	Distance	σ^2 ($Å^2$)^c	R-factor
Ag-O	3.12 ± 0.28	2.19 ± 0.01	0.0012 ± 0.0001	0.015 ± 0.001

Table S3. Data obtained from the high resolution XPS Ag 3d and O 1s spectra and from the Auger Ag MNN spectra for the Ag-doped samples.

	Ag $3d_{5/2}$ (eV)	Ag $3d_{3/2}$ (eV)	$O_α$ (eV)	$O_β$ (eV)	$M_4M_{45}M_{45}$ (eV)	$M_5M_{45}M_{45}$ (eV)	$O_β$/ ($O_α$+$O_β$)	Ag $3d_{5/2}$ + $M_4M_{45}M_{45}$ (eV)
0.7Ag	367.9	373.9	529.6	531.4	357.2	351.7	0.32	725.1
2.1Ag	368.0	374.0	529.4	531.6	356.7	352.1	0.39	724.7
7.1Ag	368.1	374.1	529.4	531.0	356.6	350.9	0.28	724.7
2.1Ag used	367.9	373.9	529.5	531.0	-	-	0.41	-

Table S4. Data obtained from the high resolution XPS $Mn2p_{3/2}$ spectra for the samples MnO_2, 0.7Ag, 2.1Ag, 7.1Ag and 2.1Ag used.

Sample	Mn^{3+} (eV)	Mn^{4+} (eV)	Mn^{3+}/ (Mn^{3+} + Mn^{4+})
MnO_2	642.1	643.7	0.54
0.7Ag	641.5	643.6	0.62
2.1Ag	641.6	643.5	0.71
7.1Ag	641.5	643.6	0.60
2.1Ag used	641.8	643.6	0.83

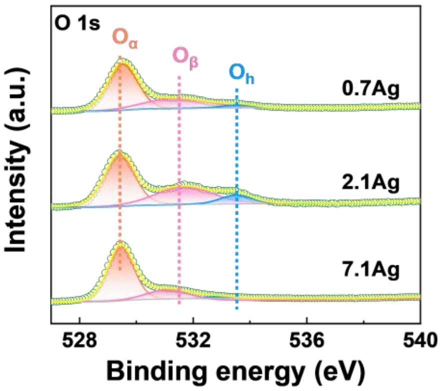

Figure S6. High-resolution O 1s XPS spectra of 0.7Ag, 2.1Ag and 7.1Ag.

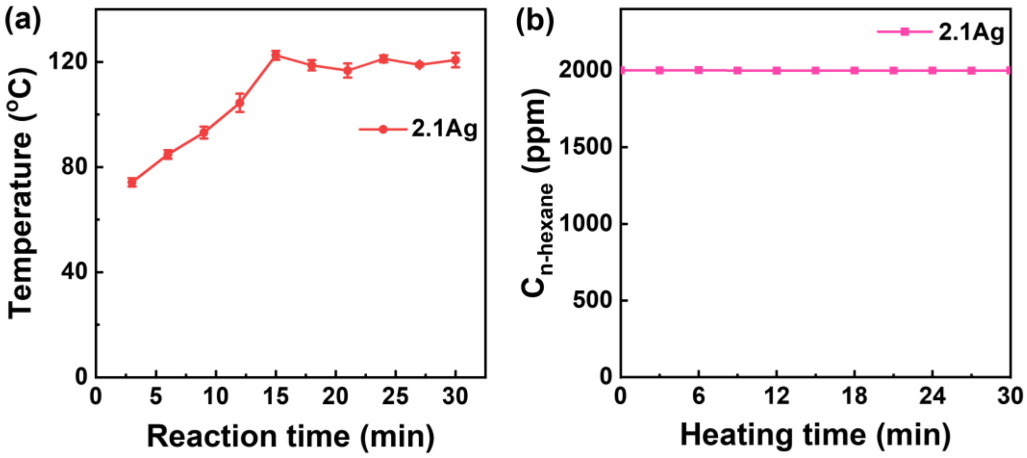

Figure S7. (a) Temperature variation of quartz tube over time during discharge; (b) Variation of n-hexane concentration with heating time using 2.1Ag at 120 °C.

3. Carbon balance and by-products

The carbon balance is calculated as followed:

$$\frac{6[\text{n-hexane}]_{\text{in}}}{1000 \times M_{\text{n-hexane}}} = \frac{6[\text{n-hexane}]_{\text{out}}}{1000 \times M_{\text{n-hexane}}} + \frac{[CO_2]_{\text{out}}}{1000 \times M_{CO_2}} + \frac{[CO]_{\text{out}}}{1000 \times M_{CO}} + [C_{\text{Others}}]_{\text{out}}$$

Where $[CO_2]_{out}$ and $[CO]_{out}$ are CO_2 outlet concentration and CO outlet concentration in mg m^{-3}, respectively; $[C_{Others}]_{out}$ is the number of carbon atom of other intermediates in the outlet gas in mol m^{-3}, $M_{\text{n-hexane}}$ is the molecular mass of n-hexane (86.18 g mol^{-1}), M_{CO_2} is the molecular mass of CO_2 (44 g mol^{-1}), M_{CO} is the molecular mass of CO (28 g mol^{-1}).

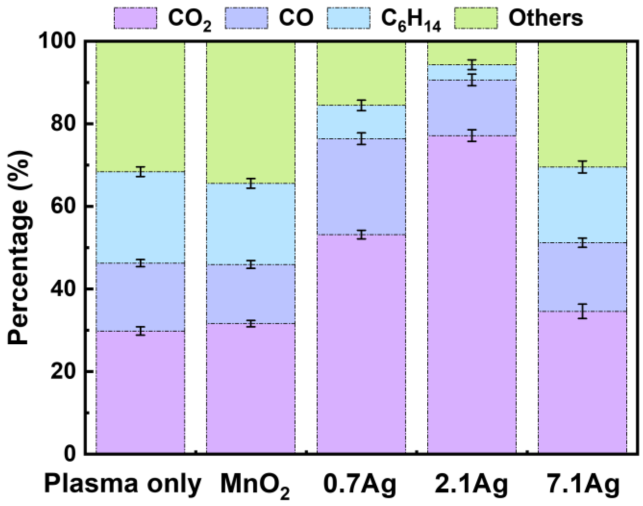

Figure S8. Carbon balance of n-hexane degradation by plasma only and the plasma-catalysis system.

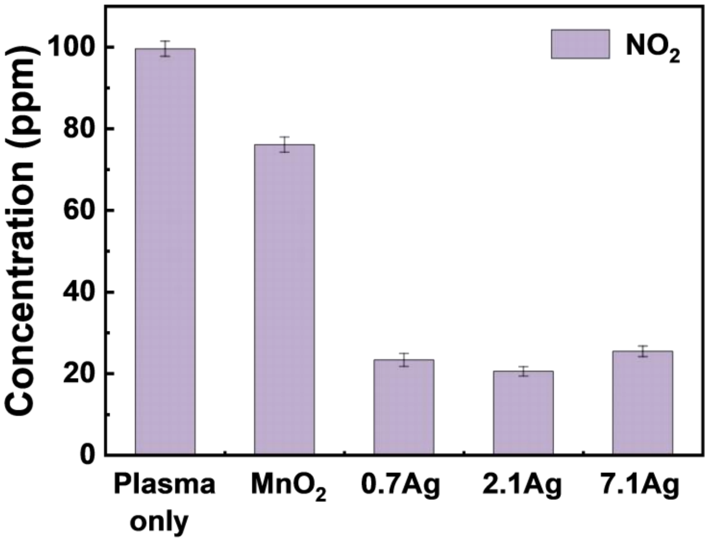

Figure S9. NO_2 concentration during n-hexane removal by plasma only and the plasma-catalysis system.

4. Characterization of used 2.1Ag after a 100 h plasma-catalysis process

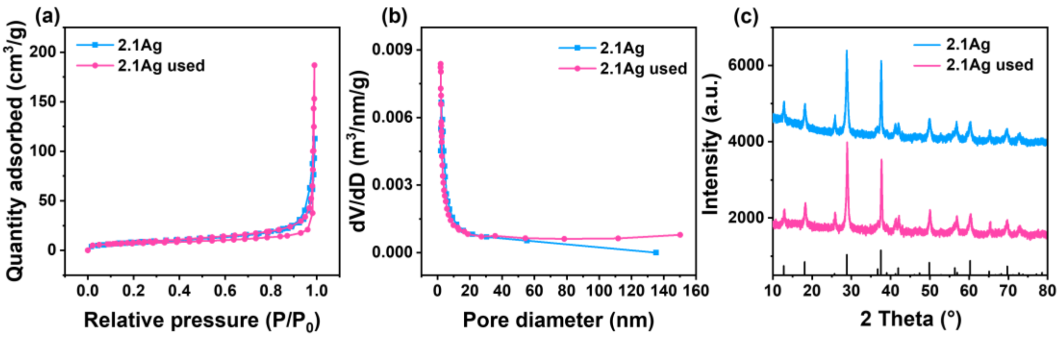

Figure S10. (a) N_2 adsorption-desorption isotherms and (b) BJH pore size of 2.1Ag and 2.1Ag used; (c) XRD spectra of 2.1Ag and 2.1Ag used.

Table S5. Comparison of the specific surface area and average pore size between fresh 2.1Ag and used 2.1Ag

Catalysts	S_{BET} (m^2 g^{-1})	D_{pore} (nm)
2.1Ag	36.5	24.4
2.1Ag used	29.4	20.9

5. Supplement of degradation pathway and the synergistic effect.

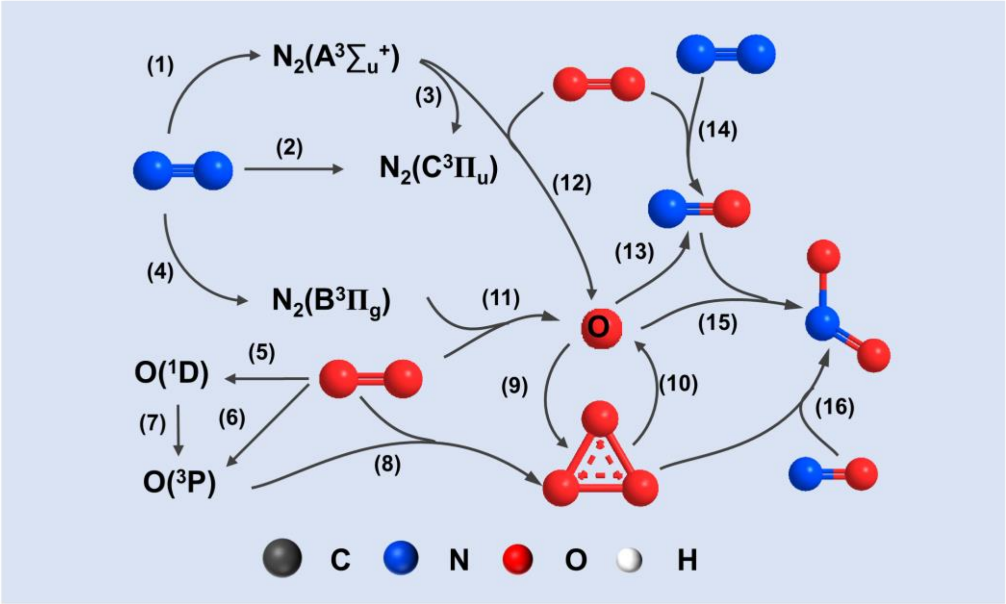

Figure S11. Diagram of the reaction mechanism between the active species in the gas phase

Table S6. Gas-phase reactions in the plasma catalysis process.

Reaction processes	Reaction equations	Number	Ref
Formation of active substances under plasma	$N_2 + e \rightarrow N_2\ (A^3\Sigma_u^+) + e$	(R1)	2, 3
	$N_2 + e \rightarrow N_2\ (C^3\Pi_u) + e$	(R2)	
	$N_2\ (A^3\Sigma_u^+) + e \rightarrow N_2\ (C^3\Pi_u) + e$	(R3)	4, 5, 6
	$N_2 + e \rightarrow N_2\ (B^3\Pi_g) + e$	(R4)	
	$O_2 + e \rightarrow O\ (^1D) + O\ (^3P) + e$	(R5)	2
	$O_2 + e \rightarrow 2O\ (^3P) + e$	(R6)	7, 8
	$O\ (^1D) \rightarrow O\ (^3P)$	(R7)	
Formation and decomposition of O_3 under plasma	$O\ (^3P) + O_2 + O_2 \rightarrow O_3 + O_2$	(R8)	9
	$O + O_2 + N_2 \rightarrow O_3 + N_2$	(R9)	10
	$O_3 + hv \rightarrow O^- + O_2$	(R10)	11, 12
	$O_2 + e \rightarrow O + O^-$		
	$O_3 + V_O \rightarrow O^{2-} + O_2$		
	$O^{2-} + O_3 \rightarrow O_2 + O_2^{2-}$		
Formation of NO_x	$N_2\ (B^3\Sigma) + O_2 \rightarrow N_2 + O + O$	(R11)	13
	$N_2\ (A^3\Sigma) + O_2 \rightarrow N_2 + O + O$	(R12)	
	$N_2 + 2O \rightarrow 2NO$	(R13)	6, 14, 15
	$N_2 + O_2 \rightarrow 2NO$	(R14)	
	$O + NO \rightarrow NO_2$	(R15)	
	$NO + O_3 \rightarrow NO_2 + O_2$	(R16)	

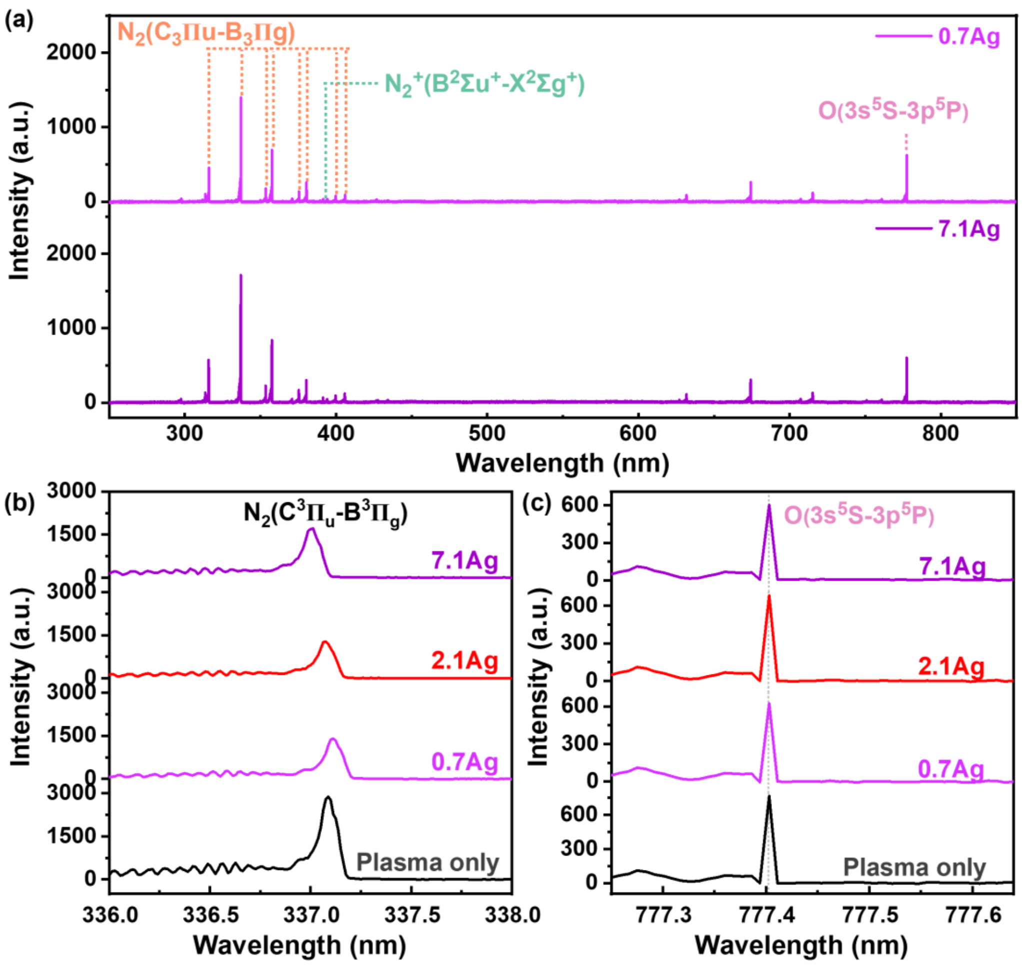

Figure S12. (a) OES spectra of a discharge sustained in n-hexane/air using 0.7Ag and 7.1Ag; (b) Zoom in of the N_2 ($C^3\Pi_u$-$B^3\Pi_g$) transition using plasma only, 0.7Ag, 2.1Ag and 7.1Ag in combination with plasma; (c) Zoom in of the O($3s^5S$-$3p^5P$) transition using plasma only, 0.7Ag, 2.1Ag and 7.1Ag in combination with plasma.

Table S7. Possible reactions in the degradation pathway of n-hexane.

Reaction equations	Process
C_6H_{14} + O* → oxygenated organic compounds	(1)
C_6H_{14} + N* → nitrogenous organic compounds	(2)
$-Mn^{4+}-O^{2-}-Mn^{4+} \rightarrow Mn^{3+}-\square-Mn^{3+} + 1/2O_2$	(3)
Intermediate products + $Ag-O^{2-}-Mn \rightarrow CO_2 + H_2O$	(4)

Table S8. Basic information of the detected gaseous intermediates by GC-MS.

Number	Name	Chemical formula	Chemical structure
1	butane	C_4H_{10}	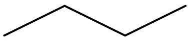
2	1-hexen-3-ol	$C_6H_{12}O$	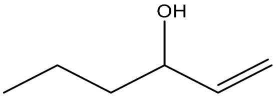
3	3-hexanone	$C_6H_{12}O$	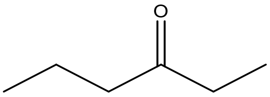
4	2-hexanone	$C_6H_{12}O$	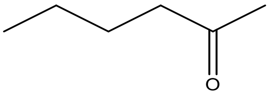
5	2-hydroxybutyrate hydrazide	$C_4H_{10}N_2O_2$	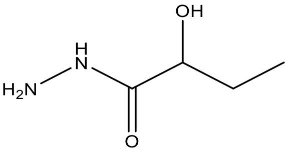
6	2-hexanol	$C_6H_{14}O$	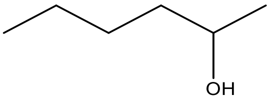
7	1-hexanoic acid	$C_6H_{12}O_2$	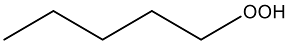
8	1-hexene	C_6H_{12}	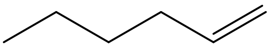
9	1,2-hexanediol	$C_6H_{14}O_2$	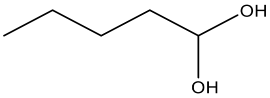
10	octane	C_8H_{18}	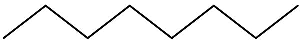
11	2,5-dimethyltetrahydrofuran	$C_6H_{12}O$	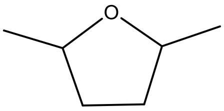
12	1-hexanol	$C_6H_{12}O$	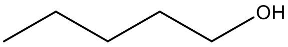
13	2,5-hexanedione	$C_6H_{10}O_2$	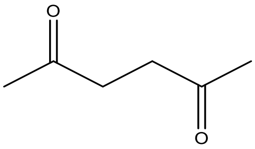
14	4-octanone	$C_8H_{16}O$	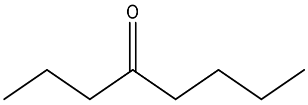

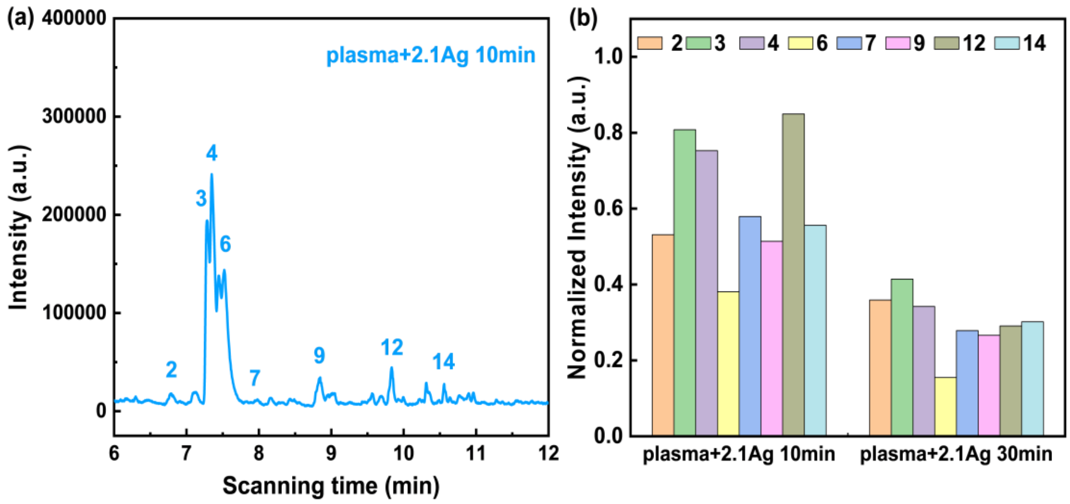

Figure S13. (a) GC-MS spectrum of the gaseous by-products of n-hexane degradation using the plasma + 2.1Ag catalytic system for 10 min; (b) Normalized intensities of the plasma + 2.1Ag catalytic system for 7 species (for numbering, see Figure 4b).

The intensities of the products were normalized using the following equation:

Normalized GC-MS intensity =

$$\frac{\text{Peak intensity (using plasma only - using plasma + 2.1Ag x min (x = 10, 30 min))}}{\text{Peak intensity using plasma only}}$$

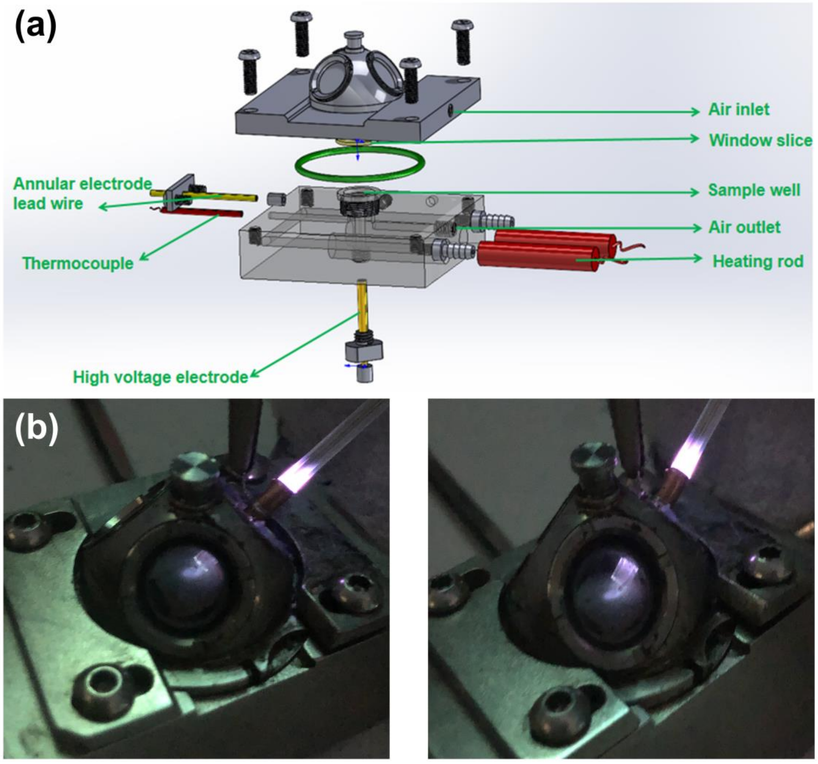

Figure S14. (a) Schematic representation of the in-situ DRIFTS device and (b) realistic view of the device in operation for plasma-catalysis reactions.

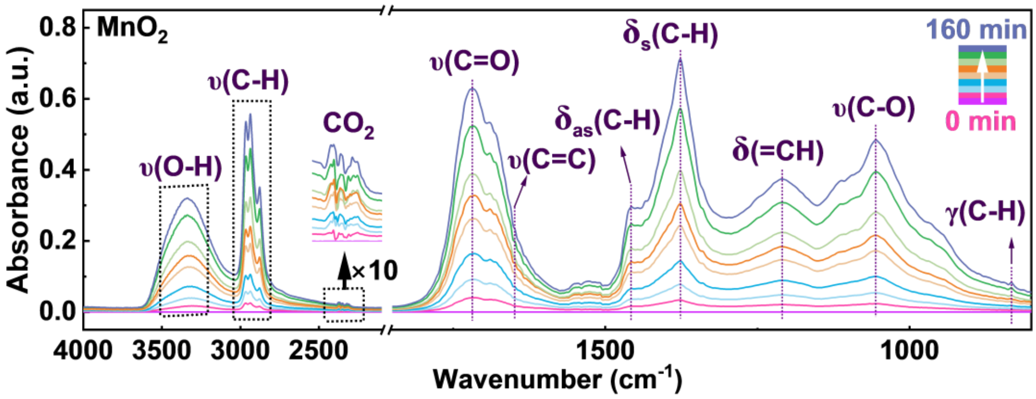

Figure S15. *In situ* DRIFTS spectra of plasma-MnO_2 for n-hexane degradation.

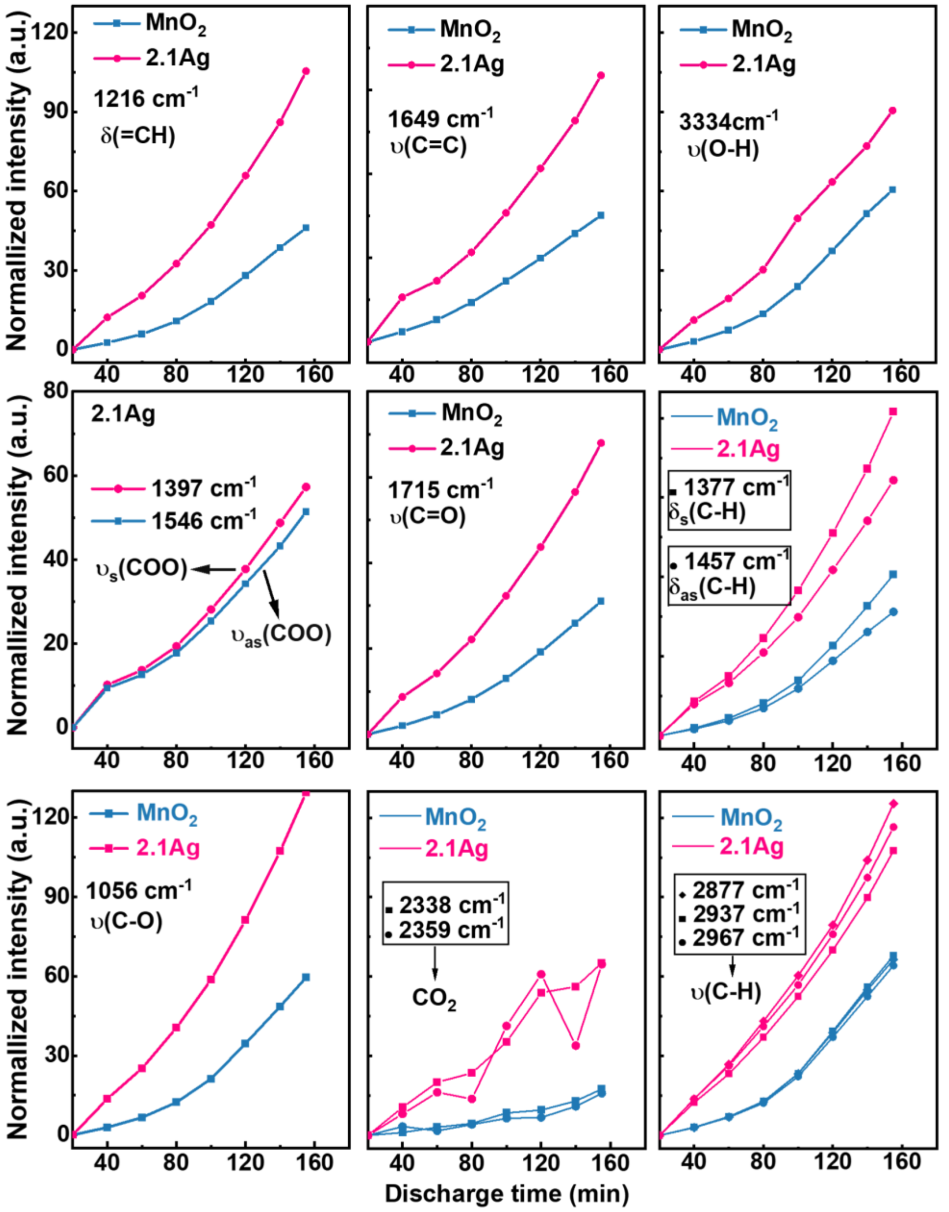

Figure S16. Normalized IR band intensities as a function of discharge time to show the evolution of different chemical bonds on MnO_2 and 2.1Ag during plasma-catalysis.

The IR band intensities were normalized by dividing the intensity values at different discharge times by the intensity obtained using the initial 20 min discharge, as shown in the following equation:

$$\text{Normalized IR intensity} = \frac{\text{Peak intensity at x min discharge } (20 < x < 160 \text{ min})}{\text{Peak intensity at 20 min discharge}}$$

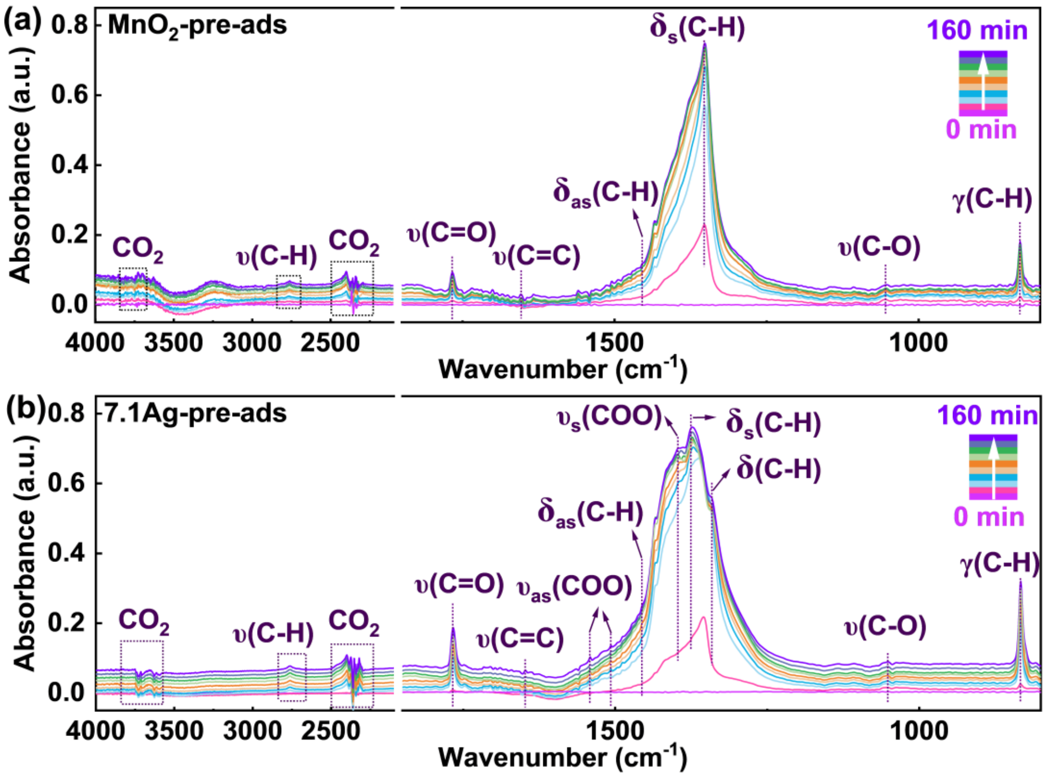

Figure S17. (a) In-situ DRIFTS spectra of plasma + MnO_2-pre-ads and (b) plasma + 7.1Ag-pre-ads for n-hexane degradation. (MnO_2-pre-ads and 7.1Ag-pre-ads: the MnO_2 and 7.1Ag sample with pre-adsorbed n-hexane was subjected to a plasma discharge in air.)

Pre-adsorption: The MnO_2, 2.1 Ag and 7.1 Ag samples were pressed and sieved to obtain a mesh size of 40 to 60. And then 50 mg of the sieved samples were placed in an *in situ*

reaction cell and exposed to 400 ppm n-hexane for 30 min, followed by continuous purging and replacement of the hexane with nitrogen for 4 h until the IR detected surface adsorbed hexane peaks stabilized, resulting in the MnO_2, 2.1 Ag and 7.1 Ag with n-hexane pre-adsorbed.

In situ DRIFTS： The catalyst sample was treated with plasma operating at 0.1 W in a gas stream of air at a rate of ~0.4 L min^{-1} (up to 160 min).

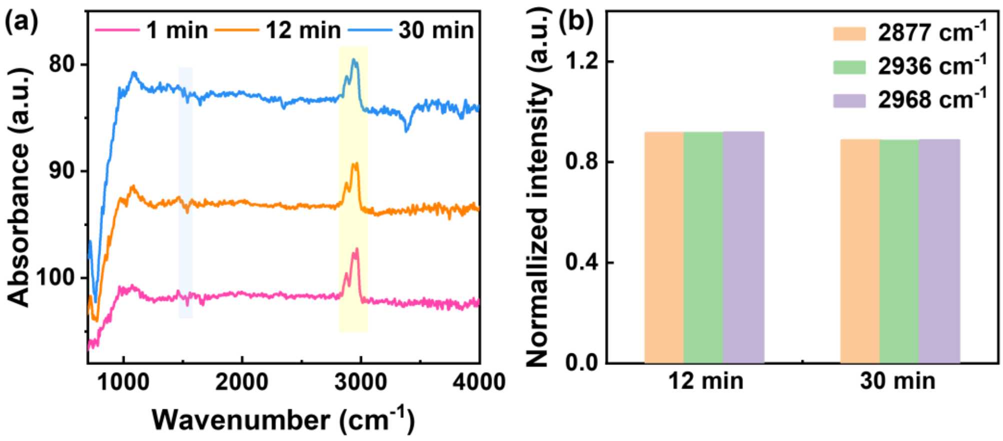

Figure S18. (a) In-situ DRIFTS spectra as a function of heating time using 2.1Ag at 120 °C for n-hexane degradation; (b) Normalized intensity of the IR bands at 2876, 2936 and 2968 cm^{-1} for 12 and 30 min of heating.

The IR band intensities were normalized by dividing the peak intensity values at different heating times by the peak intensity at the initial first min of heating, as shown in the following equation:

$$\text{Normalized IR intensity} = \frac{\text{Peak intensity at x min heating (x = 12, 30 min)}}{\text{Peak intensity at 1 min heating}}$$

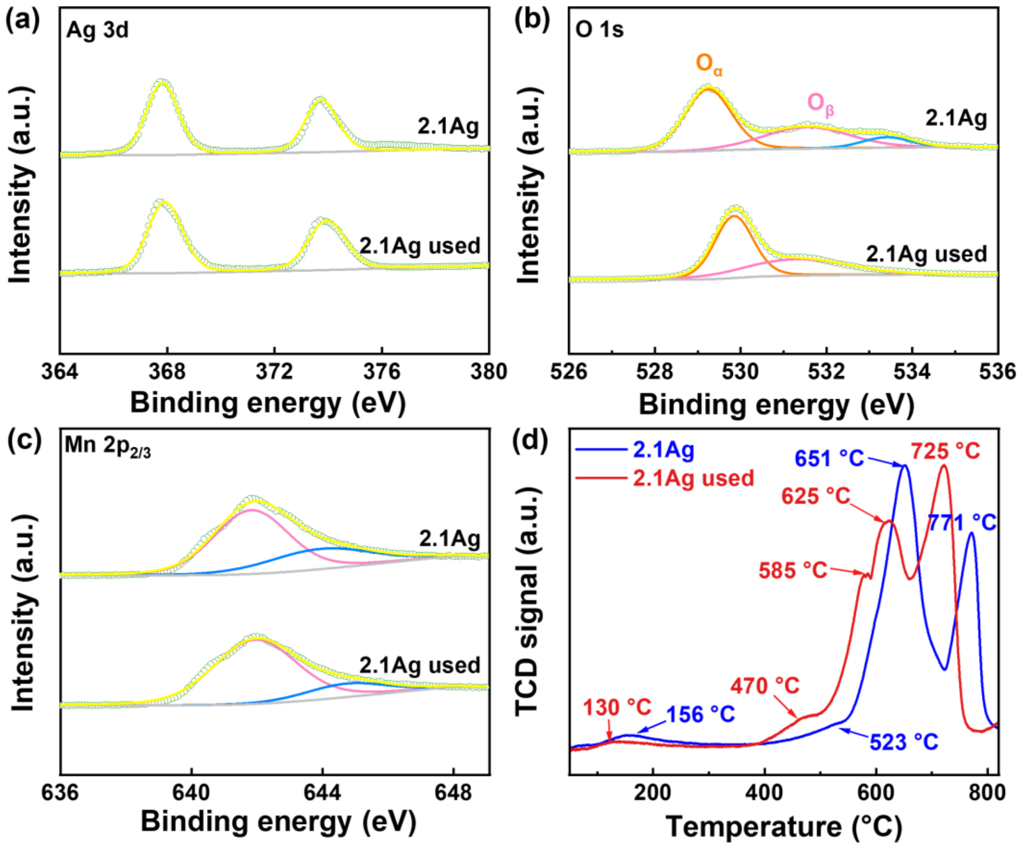

Figure S19. (a) High resolution Ag 3d, (b) O 1s and (c) Mn $2p_{2/3}$ XPS spectra of 2.1Ag and 2.1Ag used; (d) O_2-TPD curves of 2.1Ag and 2.1Ag used.

6. References

(1) Zhang, N.; Zhang, X.; Tao, L.; Jiang, P.; Ye, C.; Lin, R.; Huang, Z.; Li, A.; Pang, D.; Yan, H.; Wang, Y.; Xu, P.; An, S.; Zhang, Q.; Liu, L.; Du, S.; Han, X.; Wang, D.; Li, Y., Silver Single-Atom Catalyst for Efficient Electrochemical CO_2 Reduction Synthesized from Thermal Transformation and Surface Reconstruction. *Angew. Chem., Int. Ed.* **2021,** *60* (11), 6170-6176.

(2) Vesali-Naseh, M.; Xu, S.; Xu, L.; Khodadadi, A.; Mortazavi, Y.; Ostrikov, K., Abatement of trichloroethylene using DBD plasma. *Int. J. Mod. Phys. Conf. Ser.* **2014,** *32*, 1460344.

(3) Chen, D.; Peng, Y.; Gao, X.; Hou, Z., Effects of the N_2 to O_2 ratio in air on the removal rate and the degree of decomposition of 2-chloroethyl ethyl sulphide by atmospheric plasma. *J. Environ. Chem. Eng.* **2021,** *9* (6), 106776.

(4) Guerra, V.; Tejero-del-Caz, A.; Pintassilgo, C. D.; Alves, L. L., Modelling N_2–O_2 plasmas: volume and surface kinetics. *Plasma Sources Sci. Technol.* **2019,** *28* (7), 073001.

(5) Chen, W.; Huang, J.; Du, N.; Liu, X.-D.; Wang, X.-Q.; Lv, G.-H.; Zhang, G.-P.; Guo, L.-H.; Yang, S.-Z., Treatment of enterococcus faecalis bacteria by a helium atmospheric cold plasma brush with oxygen addition. *J. Appl. Phys.* **2012,** *112* (1), 013304.

(6) Zhou, W.; Ye, Z.; Nikiforov, A.; Chen, J.; Wang, J.; Zhao, L.; Zhang, X., The influence of relative humidity on double dielectric barrier discharge plasma for chlorobenzene removal. *J. Cleaner Prod.* **2021,** *288*, 125502.

(7) Kim, J.; Lee, H.; Huh, S.-C.; Bae, J. H.; Choe, W.; Han, D.; Park, S.; Ryu, S.; Park, S., Competitive formation of NO, NO_2, and O_3 in an air-flowing plasma reactor: A central role of the flow rate. *Chem. Eng. J.* **2023,** *468*, 143636.

(8) Herron, J. T.; Green, D. S., Chemical Kinetics Database and Predictive Schemes for Nonthermal Humid Air Plasma Chemistry. Part II. Neutral Species Reactions. *Plasma Chem. Plasma Process.* **2001,** *21* (3), 459-481.

(9) Ye, H.; Liu, Y.; Chen, S.; Wang, H.; Liu, Z.; Wu, Z., Synergetic effect between non-thermal plasma and photocatalytic oxidation on the degradation of gas-phase toluene: Role of ozone. *Chin. J. Catal.* **2019,** *40* (5), 631-637.

(10) Ye, Z.; Giraudon, J.-M.; De Geyter, N.; Morent, R.; Lamonier, J.-F., The design of MnO_x based catalyst in post-plasma catalysis configuration for toluene abatement. *Catalysts* **2018,** *8* (2), 91.

(11) Wang, B.; Chi, C.; Xu, M.; Wang, C.; Meng, D., Plasma-catalytic removal of toluene over CeO_2-MnO_x catalysts in an atmosphere dielectric barrier discharge. *Chem. Eng. J.* **2017,** *322*, 679-692.

(12) Ye, Z.; Veerapandian, S. K. P.; Onyshchenko, I.; Nikiforov, A.; De Geyter, N.; Giraudon, J.-M.; Lamonier, J.-F.; Morent, R., An in-Depth investigation of toluene decomposition with a glass Beads-Packed bed dielectric Barrier discharge reactor. *Ind. Eng. Chem. Res.* **2017,** *56* (37), 10215-10226.

(13) Asilevi, P. J.; Yi, C. W.; Li, J.; Nawaz, M. I.; Wang, H. J.; Yin, L.; Junli, Z., Decomposition of formaldehyde in strong ionization non-thermal plasma at atmospheric pressure. *Tehran. Int. J. Environ. Sci. Technol.* **2020,** *17* (2), 765-776.

(14) Morent, R.; Leys, C., Ozone Generation in Air by a DC-Excited Multi-Pin-to-Plane Plasma Source. *Ozone: Sci. Eng.* **2005,** *27* (3), 239-245.

(15) Sun, Y.; Chmielewski, A. G.; Bułka, S.; Zimek, Z.; Nichipor, H., Mechanism of decomposition of 1,4-dichlorobenzene/air in an electron beam generated plasma reactor. *Radiat. Phys. Chem.* **2007,** *76* (7), 1132-1139.
